# Sulfation of Sugarcane-Bagasse Xylan: Process Optimization, Physicochemical Properties and In Vitro Anticoagulant Activity

**DOI:** 10.3390/polym18182296

**Published:** 2026-09-19

**Authors:** Mingjun Zhang, Xuefeng Wang, Qi Wang, Jianbin Li

**Affiliations:** 1 Key Laboratory of Environmental Pollution Monitoring and Disease Control, Ministry of Education, Department of Nutrition and Food Hygiene, School of Public Health, Guizhou Medical University, Guiyang 561113, China; 2 College of Light Industry and Food Engineering, Guangxi University, Provincial and Ministerial Collaborative Innovation Center for the Sugar Industry, Engineering Research Center for the Sugar Industry and Comprehensive Utilization, Ministry of Education, Nanning 530004, China; 3 Department of Histology and Embryology, School of Basic Medicine, Guizhou Medical University, Guiyang 561113, China

**Keywords:** sugarcane, bagasse xylan, sulfation, response surface methodology, water solubility, coagulation time

## Abstract

Xylan sulfates are established anticoagulant polysaccharides, but their preparation requires balancing sulfate substitution and product recovery. Sugarcane-bagasse xylan was sulfated using sulfur trioxide–pyridine complex (SO_3_·Py), 4-dimethylaminopyridine (DMAP), and 1-ethyl-3-(3-dimethylaminopropyl) carbodiimide hydrochloride (EDCI). An entropy-weighted five-factor Box–Behnken design jointly optimized degree of substitution (DS) and isolated, DS-adjusted yield, assigning respective weights of 0.570 and 0.430. Pareto analysis identified a trade-off between increasing DS and decreasing yield, while accounting for model uncertainty produced little change in the selected optimum. Validation used adjusted reagent-to-xylan mass ratios of 0.2, 3.2, and 2.2 g/g for DMAP, SO_3_·Py, and EDCI, respectively, at 50.7 °C for 3.8 h. Three independent batches yielded DS = 1.53 ± 0.01, yield = 78.0 ± 0.1%, and a composite score of 0.792 ± 0.008, within the model’s 95% prediction interval. Operational water solubility increased from 21.2 to 96.8–130.4 mg/mL after sulfation, alongside changes in aqueous aggregation and rheology. In citrated sheep plasma, the derivatives prolonged activated partial thromboplastin time and thrombin time with increasing concentration. Responses increased across the derivative series, in which DS and the reported apparent molar mass covaried. These results provide a basis for balancing substitution and product recovery and comparing the water solubility and in vitro anticoagulant responses of the resulting derivatives.

## 1. Introduction

The valorization of lignocellulosic biomass is an important component of sustainable biorefining and resource-efficient utilization of agricultural side streams [1]. Sulfated polysaccharides derived from renewable biomass are of particular interest as non-animal polyanionic materials with anticoagulant potential [2]. Sugarcane bagasse is an abundant xylan-rich residue, and the physicochemical behavior of xylan is strongly influenced by molar mass, branching, and substituent type and distribution [3]. Introducing sulfate half-esters can increase hydration and alter interchain association while creating the anionic functionality associated with anticoagulant activity. Nevertheless, the biological response of sulfated polysaccharides is not governed by sulfate content alone; molecular weight, sulfate density and distribution, backbone architecture, and higher-order conformation can all contribute to activity [2,3].

Established sulfation methods provide a basis for preparing xylan derivatives with different substitution levels and material properties. Daus et al. obtained highly substituted xylan sulfates under homogeneous conditions using sulfur trioxide complexes [4]. Cheng et al. [5] examined how preparation conditions affected substitution, polymerization degree and anticoagulant activity in corn-stover xylan sulfates, while Chen et al. [6] reported flow synthesis and anticoagulant evaluation of sugarcane-bagasse xylan sulfate. Numerical modeling of sulfur content and product yield has also been reported for birch-wood xylan sulfation [7]. Building on these studies, the present work examines how reagent loadings, temperature and reaction time jointly affect the balance between average substitution and DS-adjusted isolated yield in a specific batch formulation. Average degree of substitution describes the overall extent of modification but does not resolve the distribution of sulfate groups along or among polymer chains. Substitution uniformity is therefore not established in the present study.

Previous work on guar gum sulfation using 4-dimethylaminopyridine (DMAP) and *N, N′*-dicyclohexylcarbodiimide (DCC) provided a rationale for examining a carbodiimide-containing formulation [8]. Here, 1-ethyl-3-(3-dimethylaminopropyl) carbodiimide hydrochloride (EDCI) was evaluated as an additive in a sulfur trioxide–pyridine complex (SO_3_·Py)/*N, N*-dimethylacetamide (DMAc) system. The literature precedent guided formulation selection; the role of EDCI in this reaction remains a working hypothesis. The formulation was evaluated through measured process responses and product properties.

This study combines a five-factor Box–Behnken design with entropy weighting and Pareto analysis to examine the balance between degree of substitution and DS-adjusted isolated yield. Three independent synthesis batches were used to test the selected practical conditions. Separately, three derivatives selected from identified design runs were compared in terms of water solubility, apparent molar-mass distributions, electrokinetic and rheological properties, and in vitro coagulation-time responses. The resulting dataset provides a basis for selecting preparation conditions and relating the measured properties of recovered sugarcane-bagasse xylan derivatives to their functional responses.

## 2. Materials and Methods

### 2.1. Materials and Chemical Reagents

Sugarcane bagasse-derived xylan (purity ≥ 95%; see Supplementary Table S1 for details) was purchased from Tianjin Heowns Biochemical Technology Co., Ltd. The standard dialysis membrane tubing (molecular weight cut-off [MWCO]: 3.5 kDa) was procured from Shanghai Yuanye Bio-Technology Co., Ltd. (Shanghai, China). Sulfur trioxide–pyridine complex (SO_3_·Py), *N, N*-dimethylacetamide (DMAc), 4-dimethylaminopyridine (DMAP), and 1-ethyl-3-(3-dimethylaminopropyl) carbodiimide hydrochloride (EDCI) were purchased from Tianjin Heowns Biochemical Technology Co., Ltd. (Tianjin, China). Isotopic solvents for spectroscopic characterization (deuterium oxide [D_2_O, 99.9 atom % D] and deuterated dimethyl sulfoxide [DMSO-*d*_6_]), alongside dextran molecular weight standards (1–670 kDa), were supplied by Sigma-Aldrich (Shanghai, China). General synthetic chemicals, including halogenated solvents and reductive agents (iodomethane, elemental iodine, potassium iodide, chloroform, dichloromethane, trifluoroacetic acid, sodium borodeuteride [NaBD_4_], and acetic anhydride), were obtained from Shanghai Macklin Biochemical Co., Ltd. (Shanghai, China). Citrated sheep plasma was obtained from Shanghai Yuanye Bio-technology Co., Ltd. (Shanghai, China). Pharmacological testing reagents, comprising sodium heparin and diagnostic hematology kits for activated partial thromboplastin time (APTT), prothrombin time (PT), and thrombin time (TT) assays, were acquired from Beijing Shidi Scientific Instrument Co., Ltd. (Beijing, China). Unless otherwise specified, all other chemical reagents utilized were of analytical grade.

### 2.2. Methylation Analysis

The methylation treatment process of xylan is shown in Figure S1. Derivatized partially methylated alditol acetates (PMAAs) were analyzed by gas chromatography–mass spectrometry (GC–MS). GC–MS was performed with a 7890A instrument gas–chromatograph (Agilent Technologies Inc., Santa Clara, CA, USA) interfaced with a 5975C mass-selective detector fitted with DB-5 MS (30 m × 0.25 mm, 0.25 μm film thickness; Agilent Technologies Inc., CA, USA) capillary column. Helium was used as the carrier gas with a flow rate at 1.5 mL·min^−1^. The split ratio was 5:1, and the solvent delay time was 6 min. The gas chromatography (GC) oven temperature was programmed at 80 °C (held for 1 min), increased to 160 °C (held for 1 min) at 20 °C·min^−1^, and finally increased to 210 °C (held for 1 min) at 20 °C·min^−1^. After operation, the temperature was held at 90 °C for 2 min. Glycosidic bond assignments were made to each peak based on relative retention times and ion spectra with reference to the Complex Carbohydrate Research Center’s database (http://www.ccrc.uga.edu accessed on 27 August 2026).

### 2.3. Preparation of Xylan Sulfate

Xylan sulfation was carried out in 50 mL of *N, N*-dimethylacetamide (DMAc) at the designated reaction temperature. For the single-factor experiments, the reaction temperature ranged from 40 to 90 °C, whereas temperatures of 40, 50, and 60 °C were employed in the Box–Behnken experimental design (Table S2). Under continuous stirring, 4-dimethylaminopyridine (DMAP), 1-ethyl-3-(3-dimethylaminopropyl) carbodiimide hydrochloride (EDCI), sulfur trioxide–pyridine complex (SO_3_·Py), and xylan were sequentially introduced into the reaction medium. All reagent-to-xylan ratios were expressed as mass ratios (g/g); specifically, the sulfating-agent-to-xylan ratio was calculated on the basis of the total mass of the SO_3_·Py complex. Upon completion of the reaction, the mixture was cooled to room temperature and neutralized with saturated aqueous sodium bicarbonate solution. The sulfated xylan product was subsequently precipitated by the addition of four volumes of anhydrous ethanol.

The crude precipitate was collected by centrifugation, redissolved in ultrapure water, and transferred to a dialysis membrane with a molecular weight cut-off (MWCO) of 3500 Da. To remove low-molecular-weight impurities, the solution was dialyzed against saturated NaHCO_3_ solution for 24 h and then extensively dialyzed against ultrapure water for an additional 48 h, with the dialysis medium replaced every 12 h. The retained fraction was subsequently lyophilized to recover the xylan sulfate product. The sulfation reaction conditions were systematically optimized using the degree of substitution (DS) and product yield as the primary evaluation criteria.

### 2.4. Determining the Degree of Substitution and Yield of Xylan Sulfate

The ester sulfate content of the functionalized biopolymer was quantified via a modified barium chloride–gelatin turbidimetric assay. Xylan sulfate (5 mg) was transferred to a stoppered test tube and hydrolyzed using 5 mL of 1 M HCl at 95 °C for 6 h. Upon cooling, a 0.2 mL aliquot of the hydrolysate was mixed with 3.8 mL of trichloroacetic acid (TCA) and 1 mL of 0.5% (*w*/*v*, weight/volume) barium chloride-gelatin reagent. Following thorough homogenization, the absorbance of the gelatin-stabilized barium sulfate (BaSO_4_) suspension was measured at 360 nm. The degree of substitution (DS) was then calculated as follows:

(1) $$\mathrm{DS}\;=\;132\;\times \;\%{\mathrm{SO}}_{4}^{2-}/(96-102\;\times \;\%{\mathrm{SO}}_{4}^{2-})$$

To account for the stoichiometric mass increase upon polymer functionalization, the xylan sulfate yield ($Y_{\mathrm{Xylan}\;\mathrm{sulfate}}$) was adjusted based on the degree of substitution (DS). The theoretical mass of the derivative, assuming quantitative recovery, was calculated using the empirically determined DS. The adjusted yield was then determined from the ratio of the purified product mass to this theoretical mass Equation (2):

(2) $$Y_{\mathrm{Xylan}\;\mathrm{sulfate}}\;=\;\frac{m_{\mathrm{product}}}{m_{0}\;\times \;\frac{132.11\;+\;\mathrm{DS}\;\times \;102.04}{132.11}}\;\times \;100\%$$

where $m_{0}$ represents the initial dry mass of the xylan feed and $m_{\mathrm{product}}$ denotes the mass of the purified, lyophilized product.

### 2.5. Optimization of Experimental Design

(1) Single-Factor Experimental Design

Separate single-factor screening series were used to examine the effects of DMAP, SO_3_·Py, and EDCI loadings, reaction temperature, and reaction time on DS and DS-adjusted isolated yield. Within each series, the factor under investigation was varied while the remaining factors were held at the conditions specified in the corresponding figure caption. These experiments provided conditional response trends to guide the selection of factor ranges for the subsequent Box–Behnken design; selected levels were not carried forward through a sequential optimization procedure. Reagent loadings were expressed as reagent-to-xylan mass ratios (g/g). SO_3_·Py denotes the complete sulfur trioxide–pyridine complex, and EDCI denotes the hydrochloride reagent specified in Section 2.3. At each factor level, DS and the yield calculated using Equation (2) were plotted separately and paired in a DS–yield panel. Point labels identify the varied factor level. Each factor level was represented by a separately prepared xylan sulfate sample. Samples obtained at nominally identical conditions in different single-factor series were prepared independently. The reported means and SDs summarize three technical determinations on each prepared sample.

(2) Optimization of Xylan Sulfate Synthesis via Integrated Entropy Weight Method-Response Surface Methodology (EWM-RSM)

The entropy weight method (EWM) was used to assign weights to DS and DS-adjusted isolated yield according to their relative dispersion across the experimental dataset. The weighted composite score was then modeled using response surface methodology. These weights should be interpreted as statistical weighting coefficients rather than measures of intrinsic process or biological importance. In this study, the degree of substitution (DS) and the yield (*Y*) of xylan sulfate were selected as the primary evaluation metrics. The weight coefficient (*W_j_*) for each metric was determined via EWM to calculate a comprehensive score ($S_{i}$), which served as the foundational metric for subsequent optimization experiments. The method described in reference [9] is used to calculate S_i_.

A five-factor, three-level Box–Behnken design (BBD) was subsequently used for joint optimization of the entropy-weighted composite score (S_i_). The factor ranges were selected with reference to the preliminary single-factor response trends. The design estimated linear, quadratic, and interaction effects within the investigated domain. The independent variables and their coded operational ranges (low [−1], center [0], high [+1]) were DMAP-to-xylan mass ratio (A: 0.15–0.25), SO_3_·Py-to-xylan mass ratio (B: 1–5), EDCI-to-xylan mass ratio (C: 1–3), reaction temperature (D: 40–60 °C), and reaction duration (E: 2–6 h) (Table S2). A total of 46 runs, including six center-point replicates, were executed in the randomized run order stored in the Design-Expert project file.

Analysis of variance (ANOVA) was subsequently performed on the experimental response data to establish the statistical significance of the models and elucidate the magnitude of individual parameter effects. The empirical relationship between the independent variables and the objective response was modeled using a second-order polynomial regression equation (Equation (3)) [10]:

(3) $$Y\;=\;\beta_{0}+\sum_{i=1}^{k}\beta_{i}x_{i}+\sum_{i=1}^{k}\beta_{ii}x_{i}^{2}+\sum_{i=1}^{k}\sum_{j=i+1}^{k-1}\beta_{ij}x_{i}x_{j}+\varepsilon$$

where Y represents the predicted comprehensive composite score; $\beta_{0}$ is the intercept constant; and $\beta_{i}$, $\beta_{ii}$, and $\beta_{ij}$ denote the linear, quadratic, and cross-product interaction coefficients, respectively. The terms $x_{i}$ and $x_{j}$ represent the coded values of the independent variables, and ε signifies the residual error.

The experimental design matrix was generated, the response surface topologies were rendered, and the subsequent polynomial regression analyses were executed utilizing Design-Expert software (v13.0.5, Stat-Ease Inc., Minneapolis, MN, USA).

(3) Validation of the optimized synthesis conditions.

To evaluate the reproducibility of the optimized process, three independent synthesis batches were prepared under the practical optimal conditions. Each batch was initiated using separately weighed xylan and freshly prepared reagents and was independently subjected to reaction, neutralization, ethanol precipitation, dialysis, and lyophilization. The degree of substitution was determined through three technical measurements for each independent batch, and the mean of the technical measurements was treated as the batch-level DS value. Product yield was independently calculated from the recovered dry mass of each batch. Results are reported as the mean ± standard deviation from three independent synthesis experiments (*n* = 3). Relative standard deviation (RSD) was calculated as 100 × standard deviation (SD)/mean.

### 2.6. Determination of Apparent Molar Mass and Distribution

The apparent weight-average molar mass (M_w,app_) and apparent number-average molar mass (M_n,app_) of the functionalized derivatives were determined via high-performance gel permeation chromatography (HPGPC) utilizing an Agilent 1260 system (Santa Clara, CA, USA) coupled with a refractive index detector (RID). Chromatographic separation was achieved using a TSK-GEL guard column (6.0 mm × 40 mm) connected in series with G-5000PWXL and G-2500PWXL analytical columns (7.8 mm × 300 mm each). Isocratic elution was performed at ambient temperature using 0.1 M NaNO_3_ as the mobile phase at a constant flow rate of 0.5 mL/min. Prior to injection (20 µL), samples were dissolved in the mobile phase to a concentration of ~1.0 mg/mL and clarified through a 0.45 µm membrane filter. Dextran standards (M_w_ ranging from 1270 to 667,800 Da; ~1.0 mg/mL) were used to assign apparent molar masses from retention times. The results are reported as dextran-equivalent apparent weight-average and number-average molar masses M_w,app_ and M_n,app_, with apparent dispersity calculated as dispersity (Đ_app_ = M_w,app_/M_n,app_). Each sample was subjected to three repeat injections of the same preparation.

### 2.7. Nuclear Magnetic Resonance Spectroscopy

For comprehensive structural elucidation, nuclear magnetic resonance (NMR) spectroscopy was employed. Prior to analysis, approximately 50 mg of the functionalized sample was subjected to three consecutive cycles of dissolution in deuterated dimethyl sulfoxide (DMSO-*d*_6_) and subsequent lyophilization. This rigorous pretreatment ensured complete deuterium exchange and the elimination of residual moisture. The fully exchanged specimen was subsequently redissolved in 0.5 mL of a DMSO-*d*_6_/D_2_O co-solvent mixture and transferred into a standard NMR tube. Spectroscopic acquisition was performed at 25 °C utilizing a 500 MHz Bruker AVANCE III HD 500 spectrometer (Bruker BioSpin GmbH, Ettlingen, Germany).

### 2.8. Fourier Transform Infrared and Ultraviolet–Visible Spectroscopy

The Fourier transform infrared (FT-IR) spectra were obtained using the KBr pellet method. Briefly, 2 mg of each sample was thoroughly mixed with 200 mg of dried KBr and finely ground in an agate mortar. The mixture was subsequently compressed into a transparent pellet and analyzed using a Fourier transform infrared spectrometer (TENSOR II, Bruker, Germany). Spectra were recorded over the range of 4000–400 cm^−1^ at a resolution of 4 cm^−1^ by accumulating 16 scans. A blank KBr pellet was used for background correction.

Native xylan and its sulfated derivatives (XS1–XS3) were dissolved in ultrapure water at 2 mg/mL. Ultraviolet–visible (UV–vis) spectra were recorded over 200–400 nm using a UV-3200 spectrophotometer (Shanghai Mapada Instruments Co., Ltd., Shanghai, China), with ultrapure water as the reference blank.

### 2.9. Iodine-Potassium Iodide (I_2_–KI) Chromogenic Assay

The I_2_-KI assay was employed to assess the degree of branching in the xylan and xylan sulfate backbones and to detect the presence of starch. Equal volumes of the sample (2 mg/mL) and the I_2_-KI reagent (0.02% I_2_ and 0.2% KI, *w*/*v*) were mixed and incubated at 25.0 ± 0.1 °C for 15 min to ensure the reaction proceeded sufficiently; the color change of the mixture was observed, and the absorbance was recorded over the wavelength range of 300–700 nm using a UV-Vis spectrophotometer [11].

### 2.10. Determination of Specific Optical Rotation and Water Solubility

The specific optical rotation of the biopolymers was measured to evaluate their stereochemical conformation. Water solutions of native xylan and xylan sulfate (10 mg/mL) were prepared by dissolving the samples in deionized water at 60 °C. Any residual insoluble fractions were removed via centrifugation at 15,000× *g* for 15 min. The clarified supernatants were then transferred into a 1-dm polarimeter tube. Specific optical rotation, ${\left[\alpha \right]}_{\lambda }^{t}$, was measured at 20 °C utilizing a WZZ-2S automatic polarimeter (Shanghai INESA Optical Instrument Co., Ltd., Shanghai, China) operating at the sodium D-line (λ = 589 nm). Measurements were performed in triplicate.

The water solubility of native xylan and its sulfated derivatives was determined following a modified protocol originally described by Zhang et al. [12]. Briefly, sample suspensions (up to 20% *w*/*v*) were vigorously stirred in deionized water at 80 °C for 24 h to ensure maximal dissolution. The resulting supersaturated mixtures were centrifuged at 15,000× *g* for 30 min. The undissolved solid residues were separated, recovered, and subsequently lyophilized.

### 2.11. Dynamic Light Scattering (DLS) and Electrophoretic Mobility Analysis

Native xylan and its sulfated derivatives (XS1–XS3) were dispersed in ultrapure water at concentrations of 0.1–10 mg/mL (0.01–1% *w*/*v*). Dynamic light scattering measurements were performed using a Zetasizer Nano-ZS analyzer (Malvern Instruments, Worcestershire, UK) equipped with a 633 nm helium–neon laser. Disposable polystyrene cuvettes (DTS0012) and a backscatter detection angle of 173° were used. The software settings were 1.53 for the material refractive index and 1.333, 0.8872 cP, and 78.5 for the dispersant refractive index, viscosity, and relative permittivity, respectively.

The instrument-reported Z-average hydrodynamic diameter and polydispersity index (PDI) were recorded. Diameters were calculated from the apparent diffusion coefficient using the Stokes–Einstein relationship and the specified water viscosity [13]. Because the same viscosity input was used across the concentration series, the reported diameters are treated as apparent values.

Electrophoretic mobility was measured separately in folded capillary cells (DTS1070) and converted to apparent ζ-potential using the Henry equation with the Smoluchowski approximation, F(ka) = 1.5, and the specified dispersant properties [14]. These values describe the electrokinetic response under the measurement conditions.

Samples were equilibrated at 25.0 ± 0.1 °C for 120 s before measurement. Each instrumental analysis comprised 10–100 automated runs, and the results were obtained from triplicate measurements.

### 2.12. Rheological Characterization

The rheological behavior of native xylan and its sulfated derivatives was systematically profiled following the foundational framework established by Zhou et al. [15]. Steady-shear flow dynamics and viscoelastic properties were quantified utilizing a HAAKE MARS 4 rheometer (Thermo Fisher Scientific, Karlsruhe, Germany) equipped with a parallel-plate geometry (P60TiL; 60 mm diameter, 0.5 mm gap). To evaluate steady-state flow behavior, varying concentrations of water sample solutions were subjected to shear rates ranging from 0.1 to 1000 s^−1^ at a rigidly controlled temperature of at 25 °C with a temperature control tolerance of ±1 °C. To ensure structural consistency and mitigate time-dependent polymer relaxation or degradation, all rheological measurements were performed in triplicate within 24 h of sample hydration.

Viscoelastic properties were evaluated via dynamic frequency sweeps, continuously monitoring the storage (*G′*) and loss (*G″*) moduli. Dynamic strain sweep measurements were carried out at 1 Hz to determine the linear viscoelastic regime (LVR) over a strain range from 0.1 to 1000% at 25 °C with a temperature control tolerance of ±1 °C. Afterwards, a 1.0% strain within the LVR was used to perform the frequency sweep test ranging from 0.1 to 100 rad/s. The loss tangent (tan *δ*), which quantifies the ratio of viscous response (energy dissipated) to elastic response (energy stored), was derived from the frequency sweep data:

(4) $$\tan\;\delta \;=\;\frac{G^{\prime \prime }}{G^{\prime }}$$

The magnitude of the loss tangent serves as a fundamental physical descriptor of the macromolecular network topology: tan *δ* < 1 indicates a predominantly elastic, gel-like continuous network, whereas tan *δ* > 1 signifies a viscous, fluid-like topological state.

### 2.13. In Vitro Anticoagulant Activity Assays

The in vitro anticoagulant activity of xylan sulfate was evaluated using the conventional activated partial thromboplastin time (APTT), prothrombin time (PT), and thrombin time (TT) assays, following the method described by Tang et al. [16]. Heparin sodium and physiological saline were used as the positive and negative controls, respectively. All assays were performed in accordance with the manufacturer’s instructions. To ensure thermal equilibration, all assay reagents were pre-equilibrated to 37 °C, and plasma–sample mixtures were pre-incubated at 37 °C for 3 min before initiation of coagulation.

For the APTT assay, plasma (45 μL) was mixed with the sample solution (5 μL) and incubated as described above. APTT reagent (50 μL) was then added, and the mixture was incubated at 37 °C for an additional 3 min. Coagulation was initiated by adding 50 μL of 25 mM CaCl_2_ pre-equilibrated to 37 °C, and the clotting time was recorded.

For the PT assay, plasma (45 μL) was mixed with the sample solution (5 μL) and pre-incubated at 37 °C for 3 min. PT reagent (100 μL), pre-incubated separately at 37 °C for 3 min, was subsequently added, and the time to clot formation was recorded.

For the TT assay, plasma (90 μL) was mixed with the sample solution (10 μL) and pre-incubated at 37 °C for 3 min. TT reagent (100 μL) was then added, and the clotting time was recorded.

All concentrations reported herein are final concentrations in the reaction mixture; 0 μg mL^−1^ denotes the shared physiological-saline control. Results are expressed as mean ± SD (*n* = 3). Each nonzero sample concentration was compared with the shared saline control using a *t*-test. Statistical significance was defined as * *p* < 0.05, ** *p* < 0.01, *** *p* < 0.001, and **** *p* < 0.0001.

### 2.14. Statistical Analysis

Data are presented as the mean ± standard deviation (SD). Standard deviations were reported to one significant digit, and the corresponding mean values were rounded to the same decimal place. Full-precision values were retained for all calculations, and rounding was applied only when reporting the final results. Statistical evaluations were performed using an independent Student’s *t*-test via SPSS software (version 26.0). Statistical significance was defined as *p* < 0.05.

## 3. Results and Discussion

### 3.1. Glycosidic Linkage Analysis

The glycosidic linkage composition and relative abundances of bagasse xylan are summarized in Table 1. Linkage assignments were determined based on the retention times and mass spectra of partially methylated alditol acetates (PMAAs) analyzed via gas chromatography–mass spectrometry (GC–MS). Three major linkage types—Araf-(1→, →4)-Xyl*p*-(1→ and →3,4)-Xyl*p*-(1→—predominated, accounting for 93.23% of the total glycan residues with a molar ratio of 0.40:5.03:0.79, respectively. Consistent with previous reports [17], the backbone of bagasse xylan is primarily composed of →4)-Xyl*p*-(1→ residues, encompassing both unsubstituted and monosubstituted (branched) xylose units. The Araf-(1→ residues were identified as the predominant non-reducing ends, likely linked directly to the backbone xylose chain at the O-3 position.

The degree of branching profoundly modulates the physicochemical and functional properties of xylan. Typically, bagasse xylan is characterized as a weakly branched arabinoxylan. Peng et al. [18] reported an arabinose-to-xylose (Ara/Xyl) ratio ranging from 0.04 to 0.09 for bagasse xylan. However, the Ara/Xyl ratio determined via GC–MS analysis may be underestimated, as terminal furanosyl residues are highly susceptible to acid-induced degradation during methylation [19]. In addition, trace structural components and potential impurities were detected but could not be definitively characterized due to their low abundance. For instance, minor residues such as →2)-Araf-(1→, →3,6) Gal*p* (1→ and →4)-Glc*p*-(1→ constituted only minor fractions; owing to the structural complexity of these side groups, their precise linkages and spatial assignments remain to be fully elucidated.

### 3.2. Sulfation of Xylan and Optimization of Conditions

#### 3.2.1. Preliminary Single-Factor Screening

**Figure 1:**
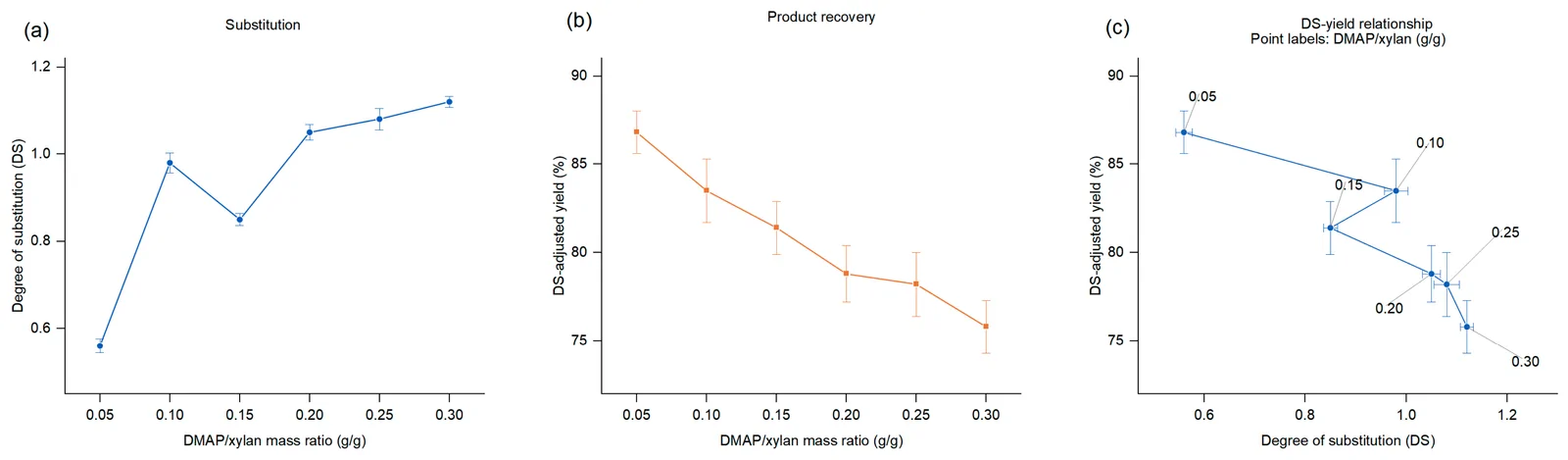
Effect of DMAP/xylan mass ratio on xylan sulfation: (**a**) degree of substitution (DS), (**b**) DS-adjusted isolated yield calculated using Equation (2), and (**c**) the corresponding DS–yield relationship. Point labels in (**c**) indicate the DMAP/xylan mass ratio in g/g. Fixed conditions were SO_3_·Py/xylan = 3.0 g/g, EDCI/xylan = 1.0 g/g, 60 °C, and 4 h, using 2.0 g xylan. Points represent means from three measurements, and error bars indicate SD; horizontal and vertical error bars in (**c**) refer to DS and yield, respectively. Dashed lines connect successive DMAP levels as visual guides and do not represent fitted curves.

**Figure 2:**
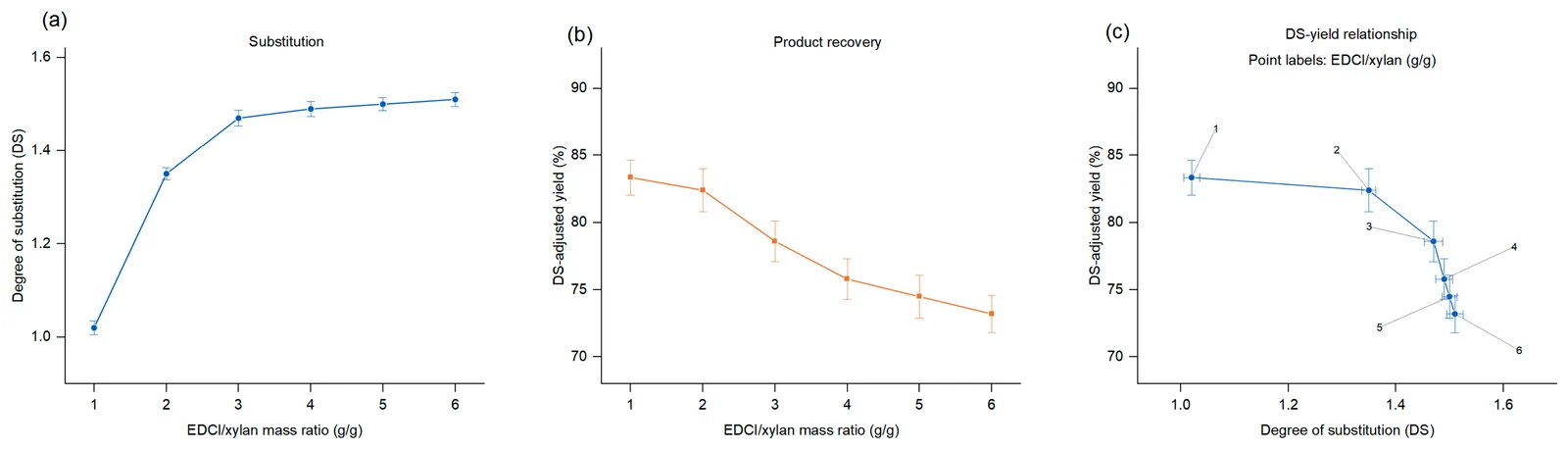
Effect of EDCI/xylan mass ratio on xylan sulfation: (**a**) degree of substitution (DS), (**b**) DS-adjusted isolated yield calculated using Equation (2), and (**c**) the corresponding DS–yield relationship. Point labels in (**c**) indicate the EDCI/xylan mass ratio in g/g. EDCI refers to the hydrochloride reagent specified in Section 2.3. Fixed conditions were DMAP/xylan = 0.10 g/g (0.20 g DMAP per 2.0 g xylan), SO_3_·Py/xylan = 3.0 g/g, 60 °C, and 4 h. Points represent means from three measurements, and error bars indicate standard deviations; horizontal and vertical error bars in (**c**) refer to DS and yield, respectively. Dashed lines connect successive factor levels in increasing order as visual guides and do not represent fitted curves.

**Figure 3:**
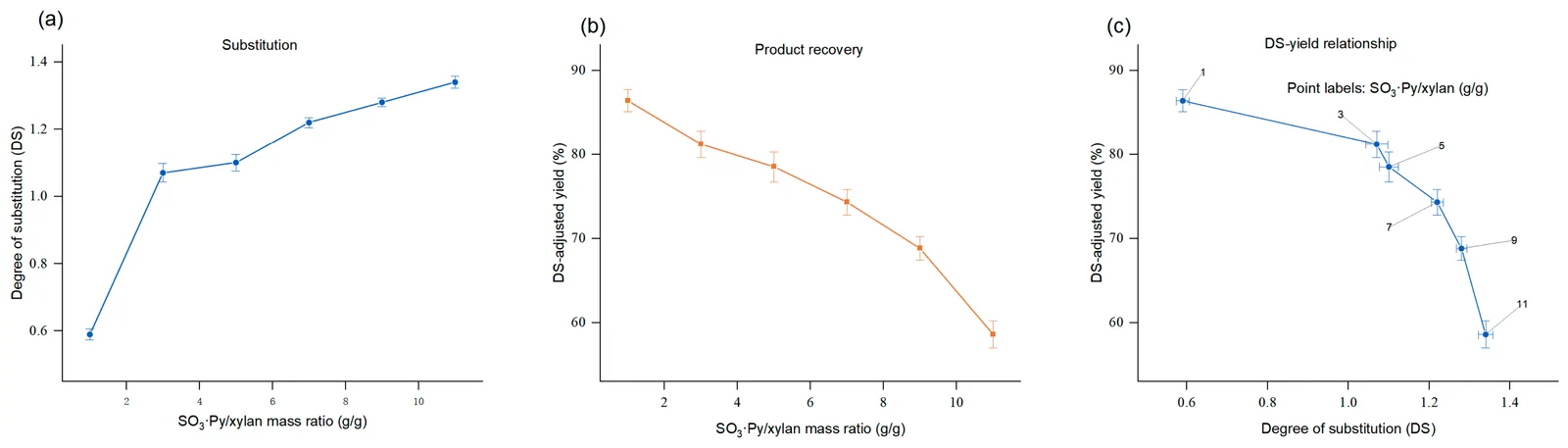
Effect of SO_3_·Py/xylan mass ratio on xylan sulfation: (**a**) degree of substitution (DS), (**b**) DS-adjusted isolated yield calculated using Equation (2), and (**c**) the corresponding DS–yield relationship. Point labels in (**c**) indicate the SO_3_·Py/xylan mass ratio in g/g, calculated using the mass of the complete sulfur trioxide–pyridine complex. Fixed conditions were DMAP/xylan = 0.10 g/g (0.20 g DMAP per 2.0 g xylan), EDCI/xylan = 1.0 g/g, 60 °C, and 4 h. Points represent means from three measurements, and error bars indicate standard deviations; horizontal and vertical error bars in (**c**) refer to DS and yield, respectively. Dashed lines connect successive factor levels in increasing order as visual guides and do not represent fitted curves.

**Figure 4:**
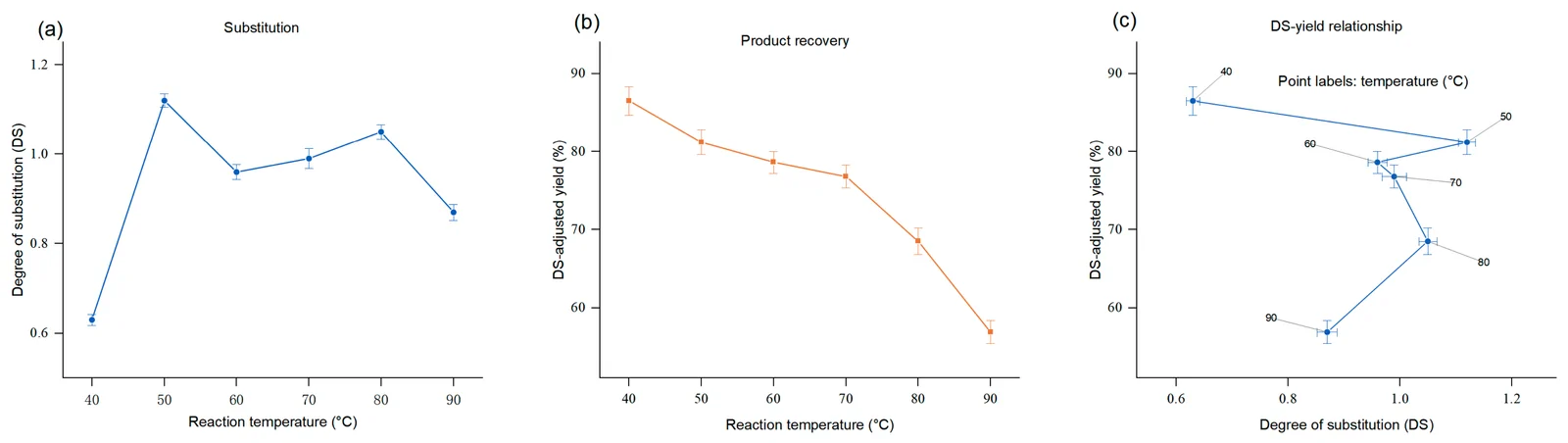
Effect of reaction temperature on xylan sulfation: (**a**) degree of substitution (DS), (**b**) DS-adjusted isolated yield calculated using Equation (2), and (**c**) the corresponding DS–yield relationship. Point labels in (**c**) indicate reaction temperature in °C. Fixed conditions were DMAP/xylan = 0.10 g/g (0.20 g DMAP per 2.0 g xylan), SO_3_·Py/xylan = 3.0 g/g, EDCI/xylan = 1.0 g/g, and 4 h. Points represent means from three measurements, and error bars indicate standard deviations; horizontal and vertical error bars in (**c**) refer to DS and yield, respectively. Dashed lines connect successive factor levels in increasing order as visual guides and do not represent fitted curves.

**Figure 5:**
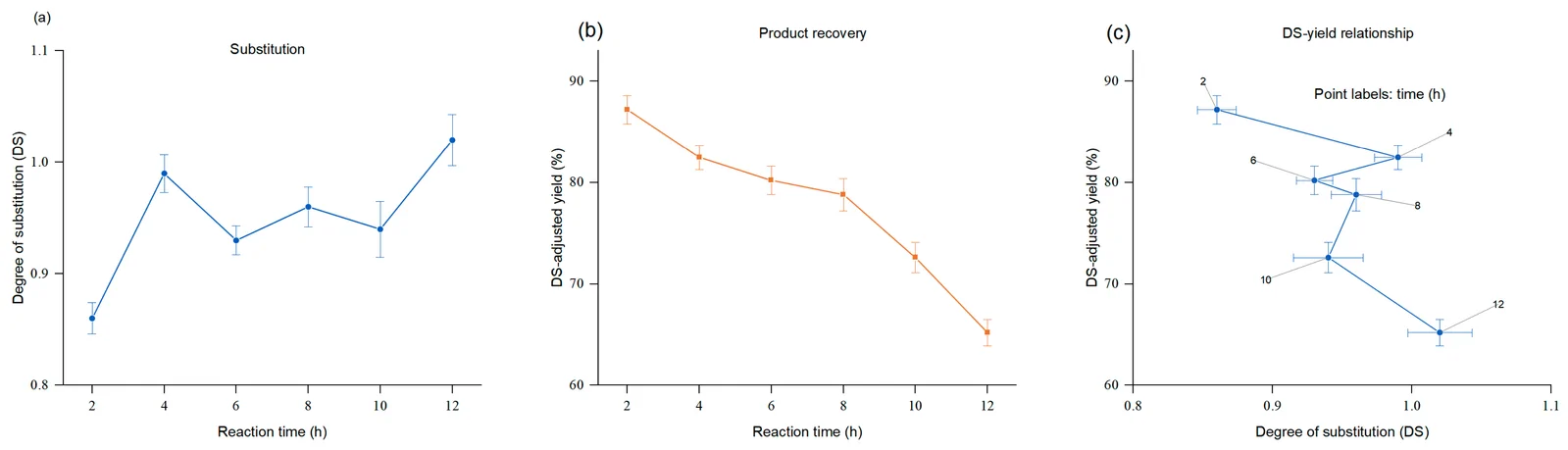
Effect of reaction time on xylan sulfation: (**a**) degree of substitution (DS), (**b**) DS-adjusted isolated yield calculated using Equation (2), and (**c**) the corresponding DS–yield relationship. Point labels in (**c**) indicate reaction time in h. Fixed conditions were DMAP/xylan = 0.10 g/g (0.20 g DMAP per 2.0 g xylan), SO_3_·Py/xylan = 3.0 g/g, EDCI/xylan = 1.0 g/g, and 60 °C. Points represent means from three measurements, and error bars indicate standard deviations; horizontal and vertical error bars in (**c**) refer to DS and yield, respectively. Dashed lines connect successive factor levels in increasing order as visual guides and do not represent fitted curves.

Both DS and DS-adjusted isolated yield were evaluated to assess sulfate substitution alongside product recovery. Previous studies have also considered DS and yield together when evaluating polysaccharide sulfation conditions [20,21]. The responses in Figure 1, Figure 2, Figure 3, Figure 4 and Figure 5 are interpreted within their respective fixed conditions. Nominally identical reference conditions in different series correspond to separately prepared samples. These screening results informed the BBD ranges and were not treated as a combined set of optimized synthesis conditions. The DS–yield panels in Figure 1, Figure 2, Figure 3, Figure 4 and Figure 5 pair the two responses obtained at each factor level. Because the yield calculation includes DS in the theoretical product mass, the two plotted quantities share a mathematical dependence.

(1) Effect of DMAP loading on degree of substitution and DS-adjusted yield

Under the fixed conditions specified in Figure 1, DS showed an overall increase with DMAP loading. DS increased from 0.56 at DMAP/xylan = 0.05 g/g to 0.98 at 0.10 g/g, decreased to 0.85 at 0.15 g/g, and then increased to 1.05 at 0.20 g/g (Figure 1a,b). Further increases in loading raised DS to 1.12 at 0.30 g/g. The DS-adjusted isolated yield decreased from 86.8% to 75.8% across the tested range. Between 0.20 and 0.30 g/g, DS increased by 0.07 while yield decreased from 78.8% to 75.8%; the additional increase in DS was smaller than that over the lower loading range [22]. A DMAP/xylan ratio of 0.20 g/g was selected as the centre level for the subsequent BBD, which examined the range of 0.15–0.25 g/g (Table S2).

Mechanistically, DMAP acts as a nucleophilic catalyst by reacting with SO_3_·Py to form a highly reactive *N*-sulfonylpyridinium intermediate [23]. The hydroxyl groups of xylan subsequently attack this intermediate to form sulfate esters while regenerating DMAP. This catalytic pathway enhances the reactivity of xylan hydroxyl groups and promotes sulfation. The proposed mechanism is illustrated in Figure S2.

(2) Effect of EDCI loading on degree of substitution and DS-adjusted yield

Under the fixed conditions specified in Figure 2, including DMAP/xylan = 0.10 g/g, increasing EDCI/xylan from 1.0 to 3.0 g/g increased DS from 1.02 to 1.47, while the DS-adjusted yield decreased from 83.35% to 78.6%. At 2.0 g/g, DS was 1.35 and yield was 82.4%. Further increasing EDCI/xylan from 3.0 to 6.0 g/g produced a smaller increase in DS, from 1.47 to 1.51, while yield fell to 73.2%, possibly due to the formation of *N*-acylurea-type byproducts from excess activated intermediates [24]. Figure 2c shows the paired responses at the six EDCI levels. The EDCI/xylan ratio of 2.0 g/g was used as the centre level in the Box–Behnken design.

Under anhydrous conditions, EDCI may undergo ligand exchange with SO_3_·Py to form a zwitterionic EDCI–SO_3_ adduct with release of pyridine [25]. Subsequent nucleophilic attack by xylan hydroxyl groups at the electrophilic sulfur center, followed by proton transfer and N–S bond cleavage, may generate xylan sulfate esters while regenerating EDCI. The proposed pathway is shown in Figure S3.

(3) Effect of SO_3_·Py loading on degree of substitution and DS-adjusted yield

The effect of SO_3_·Py loading was examined at the fixed reference conditions specified in Figure 3, including DMAP/xylan = 0.10 g/g. DS increased from 0.59 to 1.07 as SO_3_·Py/xylan increased from 1.0 to 3.0 g/g by enhancing the probability of reaction between xylan hydroxyl groups and electrophilic sulfur centers. Further increases in loading raised DS to 1.34 at 11.0 g/g, while yield decreased from 81.2% at 3.0 g/g to 58.6% at 11.0 g/g, likely because most accessible hydroxyl groups had been consumed and the remaining sites were sterically hindered. Excess SO_3_·Py may also increase reaction-mixture viscosity and promote side reactions with DMAP and EDCI, further limiting sulfation efficiency, consistent with Liu et al. [26]. In contrast, the product yield decreased progressively, possibly due to acid-induced depolymerization of the xylan backbone. A centre level of 3.0 g/g was used in the subsequent BBD, which examined the range of 1.0–5.0 g/g.

At the molecular level, sulfate transfer may proceed through a transition state involving weak N–S and O–S interactions via either a concerted or a loose “exploded” pathway. The sulfation efficiency therefore depends strongly on the nucleophilicity of xylan hydroxyl oxygens, with more electron-rich hydroxyl groups preferentially attacking the electrophilic sulfur center to form sulfate esters [27].

(4) Effect of reaction temperature on degree of substitution and DS-adjusted yield

Under fixed conditions (6.0 g SO_3_·Py, 2.0 g xylan, 0.2 g DMAP, 2.0 g EDCI, and 4 h), the effect of reaction temperature on the degree of substitution (DS) and yield was investigated (Figure 4). Within the temperature series, the highest measured DS was 1.12 at 50 °C under the fixed conditions specified in Figure 4, reflecting enhanced molecular mobility and reactivity of xylan hydroxyl groups. Further temperature increases slightly reduced the DS, likely due to thermal degradation of the polysaccharide backbone and an unfavorable shift in the equilibrium of the exothermic sulfation reaction. A similar temperature-dependent decrease in DS and yield has been reported by Wang et al. [8].

The xylan sulfate yield decreased progressively with increasing temperature. Under the acidic and dehydrating conditions generated by SO_3_·Py, elevated temperatures accelerate dehydration, glycosidic bond cleavage, and chain scission, thereby promoting xylan depolymerization and reducing product recovery.

(5) Effect of reaction time on degree of substitution and DS-adjusted yield

Under the conditions specified in Figure 5, DS increased from 0.86 at 2 h to 0.99 at 4 h. At 6–12 h, the measured DS values ranged from 0.93 to 1.02, with the highest value observed at 12 h. The DS-adjusted yield decreased from 87.2% at 2 h to 82.5% at 4 h and 65.2% at 12 h. Extending the reaction from 4 to 12 h therefore increased DS by only 0.03 while reducing yield by 17.3 percentage points. These observations informed the selection of 4 h as the BBD centre level, although the highest measured DS in this series occurred at 12 h.

The xylan sulfate yield also decreased with prolonged reaction time. Extended exposure to the reaction medium markedly reduced product recovery, whereas DS fluctuated within a relatively narrow range after 4 h. This trend is consistent with the findings of Wang et al. [28] for sulfated *Artemisia sphaerocephala* polysaccharide.

#### 3.2.2. Response Surface Analysis Box–Behnken Design

Building upon the preliminary single-factor analyses, the sulfation process was statistically optimized utilizing a five-variable, three-level Box–Behnken design (BBD). The experimental matrix evaluated five independent process parameters (Table S2): DMAP-to-xylan mass ratio (A), SO_3_·Py-to-xylan mass ratio (B), EDCI-to-xylan mass ratio (C), reaction temperature (D), and reaction time (E). Joint optimization was performed using an entropy-weighted composite score that balanced DS and DS-adjusted isolated yield. The complete experimental matrix and the corresponding empirical responses are summarized in Table S3.

**Figure 6:**
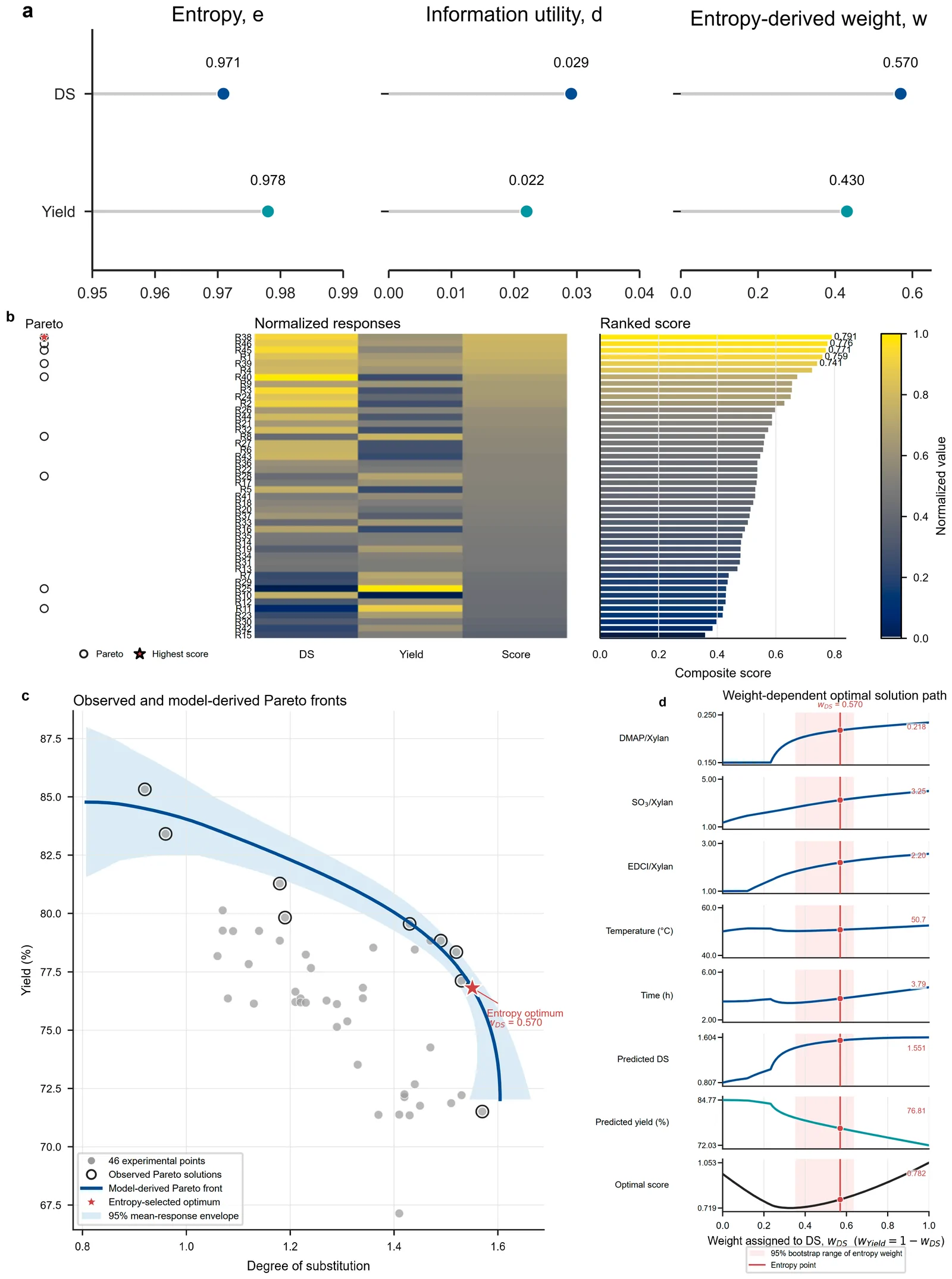
Entropy-based multi-response evaluation and continuous response-surface optimization of xylan sulfation. (**a**) Entropy, information utility and objective weights calculated from the experimental degree of substitution (DS) and isolated yield. Lower entropy and correspondingly greater information utility assigned a moderately higher weight to DS ($w_{DS}=0.570$) than to yield ($w_{Yield}=0.430$). (**b**) Run-level profiles of min–max-normalized DS, yield and entropy-weighted composite score for the 46 Box–Behnken design experiments. Runs are ordered by decreasing composite score; open circles indicate experimentally observed Pareto solutions, and the red star identifies the highest observed composite score. (**c**) Experimental DS–yield combinations (grey circles), experimentally observed non-dominated solutions (open circles), and the continuous Pareto front obtained by optimizing the fitted five-factor quadratic response surfaces over the experimental factor domain (blue line). The red star denotes the continuous entropy-selected optimum at ($w_{DS}=0.570$). Blue shading represents the pointwise 95% bivariate confidence envelope for the estimated mean responses conditional on the nominal Pareto-optimal settings. (**d**) Weight-dependent continuous optimum path. For each value of ($w_{DS}$) from 0 to 1, the DMAP/Xylan, SO_3_·Py /Xylan and EDCI/Xylan ratios, reaction temperature and reaction time were re-optimized within the experimental factor bounds. The corresponding model-predicted DS, yield and normalized weighted score are shown. The red vertical line marks the entropy-derived weight, and the pale-red band denotes its 95% fixed-design paired-residual bootstrap interval.

Figure 6 establishes the decision basis for the multi-response optimization of sugarcane-bagasse xylan sulfation. Entropy weighting objectively assigns relative importance according to the information dispersion of each response, whereas simultaneous-response and Pareto optimization provide established frameworks for resolving competing process objectives [29]. In the present dataset, the entropy-derived weights for DS (0.570) and isolated yield (0.430) place slightly greater emphasis on sulfation while retaining substantial consideration of product recovery. Within the fitted experimental domain, the Pareto front indicates a trade-off between predicted DS and DS-adjusted isolated yield. The entropy-selected solution represents one balance between these responses under the stated weighting scheme. Because the yield calculation incorporates DS, these responses are mathematically coupled; their inverse relationship should not, by itself, be interpreted as evidence of backbone degradation. Accordingly, the selected conditions (DMAP/xylan = 0.218, SO_3_·Py/xylan = 3.246, EDCI/xylan = 2.198, 50.73 °C, and 3.79 h) provide predicted values of DS = 1.551, yield = 76.81%, and composite score = 0.782. The smooth weight-dependent solution path further indicates that this operating point lies within a stable compromise region rather than being driven by an individual centre-point replicate.

Figure S4 evaluates the statistical structure, global factor importance, and internal predictive adequacy of the quadratic composite-response model. Second-order response-surface models are particularly suitable for estimating linear effects, interactions, curvature, and stationary operating conditions in multivariable processes. The significant linear terms and uniformly negative quadratic effects indicate pronounced response curvature, while the significant interaction terms demonstrate that the process factors do not act independently. Variance-based Sobol analysis, which distinguishes first-order contributions from total effects involving interactions [30], ranks the factors as DMAP/xylan (A) ≈ temperature (D) > SO_3_·Py/xylan (B) > EDCI/xylan (C) > reaction time (E). Although A and D are the principal global controls, B has the largest interaction contribution ($S_{\mathrm{Ti}}\;-\;S_{i}\;=\;0.0371$), with 98.85% attributable to its significant pairwise interactions. Leave-one-out prediction, an established cross-validatory approach to assessing predictive performance [31], yielded ($R_{LOO}^{2}$ = 0.933, root mean square error (RMSE) = 0.0285; *n* = 46), supporting application of the model within the investigated domain.

Figure S5 clarifies how the principal factor interactions reshape the composite-response surface. Previous studies have shown that sulfating-agent dosage, temperature, reaction time, and solvent environment substantially affect the substitution level and structural integrity of xylan sulfates [6,32]. In the present model, the positive BD (=0.042, *p* < 0.001), AB (β = 0.031, *p* = 0.008), and BE (β = 0.029, *p* = 0.011) interactions show that the contribution of SO_3_·Py loading depends strongly on temperature, DMAP loading, and reaction time. Conversely, the negative CD (β = −0.027, *p* = 0.017) and DE (β =−0.023, *p* = 0.044) interactions indicate diminishing composite-response gains when EDCI loading, temperature, and reaction time are increased concurrently. The closed contours and interior maxima therefore favour coordinated, moderate factor levels over extreme conditions.

Figure S6 separates the response-specific behaviour underlying the multi-objective solution. Earlier investigations have demonstrated that xylan-sulfation severity can influence DS together with product yield, polymerization degree, or molecular integrity, thereby requiring a balance between extensive substitution and preservation of the recovered derivative [6,32]. Consistent with this process context, the DS-oriented optimum is displaced towards greater SO_3_·Py loading and a somewhat higher temperature, whereas the yield-oriented optimum favours substantially lower SO_3_·Py loading and moderate thermal conditions. The composite optimum lies between these response-specific solutions, demonstrating how the selected process preserves a high sulfation level while limiting the associated loss in isolated product recovery. The arrows indicate the direction of this compromise. Moreover, the plotted markers are two-dimensional projections of the corresponding five-factor optima, with the remaining factors fixed at the composite settings.

**Figure 7:**
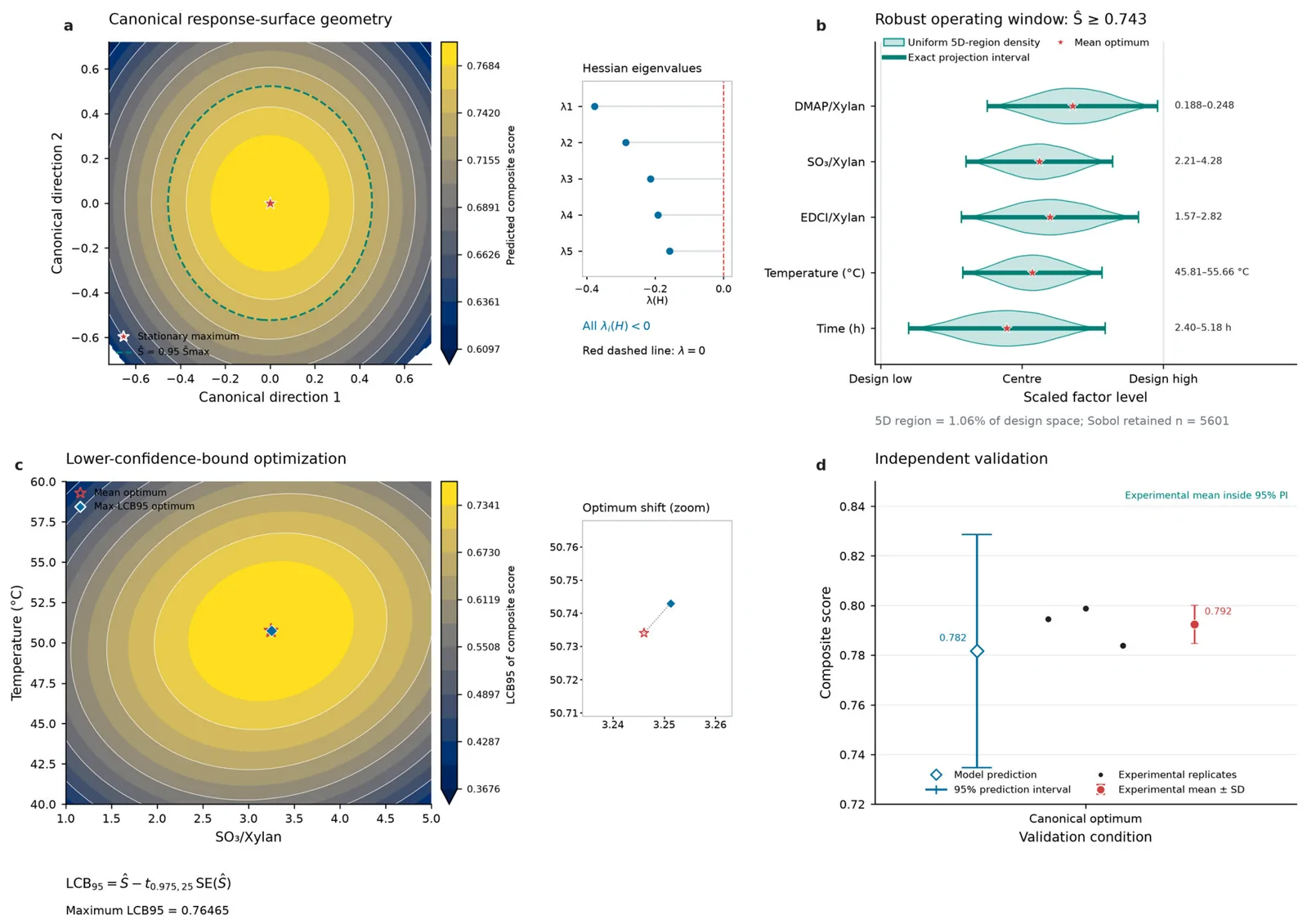
Canonical response-surface geometry, robust operating window, uncertainty-aware optimization, and independent validation of xylan sulfation. The composite score (S) integrates the normalized degree of substitution (DS) and isolated yield and was modelled using a full quadratic response surface based on a five-factor, 46-run Box–Behnken design. (**a**) Canonical analysis confirms a strictly concave response surface with a well-defined interior maximum. (**b**) The five-dimensional near-optimal region defines the process windows capable of maintaining at least 95% of the predicted maximum score. (**c**) Lower-confidence-bound optimization identifies the operating condition that maximizes predicted performance after accounting for uncertainty in the estimated mean response. (**d**) Three independent validation experiments confirm that the experimental composite scores are consistent with the model prediction and its 95% prediction interval.

Figure 7 evaluates the local curvature, operational robustness, uncertainty sensitivity, and experimental reproducibility of the selected solution. Canonical analysis of a second-order response surface provides a formal basis for distinguishing a maximum from a minimum, saddle point, or ridge and for defining an appropriate near-optimal operating region [33]. In the present model, the uniformly negative Hessian eigenvalues confirm that the stationary point is a strict model-predicted maximum. The region retaining at least 95% of the maximum response occupies only 1.06% of the design space, indicating a defined but practically usable operating neighbourhood in which all five factors must remain jointly coordinated. Lower-confidence-bound optimization produces an almost coincident solution, demonstrating limited displacement after model uncertainty is incorporated. For practical implementation, the model-predicted optimal conditions were adjusted to DMAP/xylan, SO_3_·Py/xylan, and EDCI/xylan mass ratios of 0.2, 3.2, and 2.2 g/g, respectively, a reaction temperature of 50.7 °C, and a reaction time of 3.8 h. Three independent validation experiments were performed under these conditions, and the results are presented in Table 2. The experimental composite score (0.792 ± 0.008, *n* = 3, RSD < 1%, Table 2) falls within the model’s 95% prediction interval, supporting the reproducibility and local predictive reliability of the optimized process.

### 3.3. Apparent Molar-Mass Distribution

**Figure 8:**
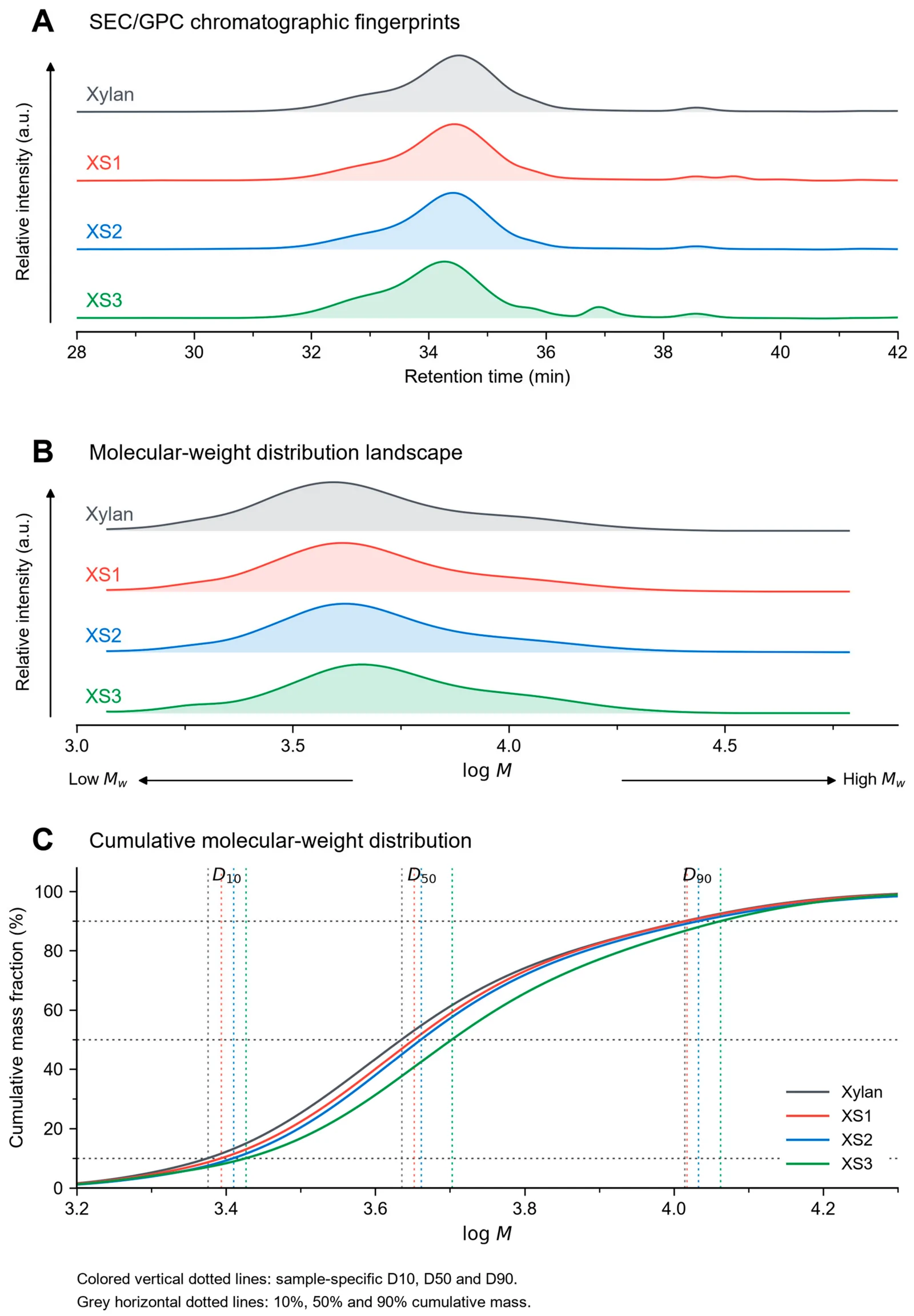
Dextran-equivalent apparent molar-mass distributions of native xylan and xylan sulfate (XS) derivatives XS1 (DS = 0.92), XS2 (DS = 1.24), and XS3 (DS = 1.57). (**A**) Vertically offset HPGPC chromatographic profiles as a function of retention time. (**B**) Normalized apparent molar-mass distributions calculated using the calibration equation $\log_{10}M=-0.2641t+12.71$, where M is expressed in Da and t in min. (**C**) Cumulative mass-fraction distributions and the corresponding $D_{10}$, $D_{50}$, and $D_{90}$ values, defined as the apparent molar masses below which 10%, 50%, and 90% of the detected polymer mass occurred, respectively. Panels (**B**,**C**) were calculated over the principal polymer-elution interval of 30.0–36.5 min. Curves in panels (**A**,**B**) were normalized separately and vertically offset; therefore, their peak heights should not be interpreted as differences in sample concentration.

XS1, XS2, and XS3 were selected from Runs 25, 41, and 40, respectively, to compare derivatives with different measured DS values (0.92, 1.24, and 1.57). Their preparation conditions and yields are reported in Section 2.3 and Table S3. High-performance gel permeation chromatography (HPGPC) was used to compare the dextran-equivalent apparent molar-mass distributions of native xylan and the selected derivatives (Figure 8). Native xylan had an apparent weight-average molar mass (M_w,app_) of 5559 Da and a dispersity (Đ) of 1.42. The corresponding values were 5790 Da and 1.41 for XS1, 5917 Da and 1.39 for XS2, and 6295 Da and 1.38 for XS3. These values indicate a numerical increase in apparent molar mass across the selected series.

The chromatographic profiles describe the fraction that dissolved in the mobile phase, passed through the sample filter, and eluted from the columns. Because retention times were converted to molar masses using dextran standards, the results depend on the relationship between hydrodynamic volume and molar mass. The introduced sulfate groups convert xylan into a polyelectrolyte and may alter chain hydration and hydrodynamic conformation. Although the 0.1 M NaNO_3_ mobile phase used here is expected to reduce electrostatic effects during SEC, their complete suppression was not independently established, as demonstrated for hydroxypropyl xylans by Pitkänen et al. [34]. Although 0.1 M NaNO_3_ was used to reduce electrostatic effects, differences in conformation and chromatographic interactions may still contribute to the apparent values.

For an idealized sodium xylan sulfate, the repeat-unit molar mass is approximately 132.11 + 102.04DS g mol^−1^. If the native value were an absolute molar mass and chain length remained unchanged, the three DS values would imply molar masses of approximately 9.51, 10.88, and 12.30 kDa. Therefore, the introduction of sulfate groups alone does not demonstrate that the chain length was maintained. Sulfation increases the mass of the substituted repeat unit but may simultaneously cause glycosidic-chain cleavage; decreases in DP or molar mass during sulfation have been reported for xylan and other lignocellulosic polysaccharides [5,35]. Accordingly, the present results are interpreted as sulfation-associated changes in SEC elution and apparent molar-mass distribution.

### 3.4. ^1^H NMR Spectroscopy

**Figure 9:**
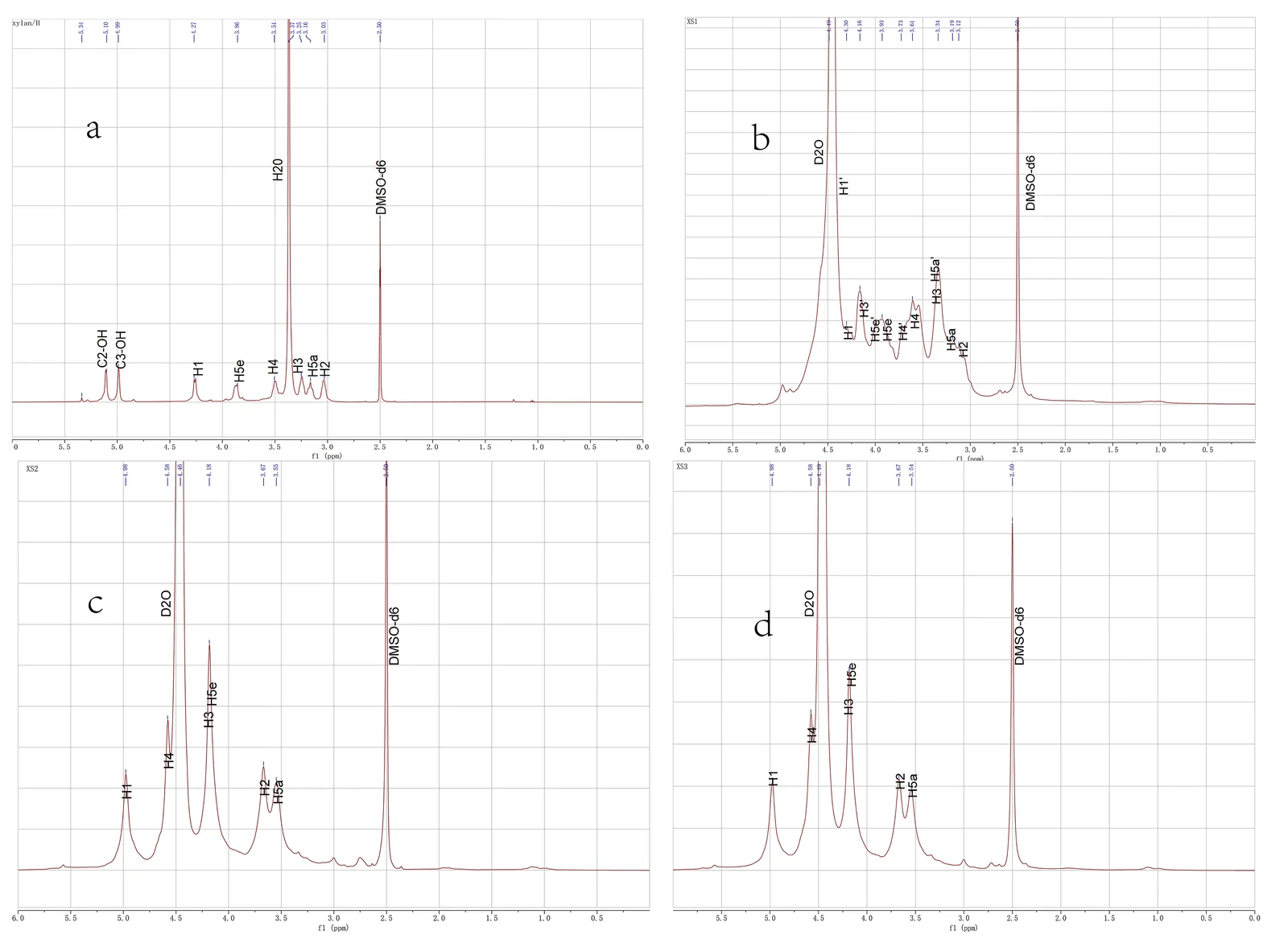
^1^H NMR spectra of native and sulfated xylans. Profiles (**a**–**d**) correspond to native xylan (**a**) and its sulfated derivatives XS1 (**b**), XS2 (**c**), and XS3 (**d**), respectively.

Figure 9 compares the ^1^H NMR spectra of native xylan and its sulfated derivatives, XS1–XS3. The spectra show changes in the anomeric and ring-proton regions following sulfation. Assignments were made with consideration of signal overlap and the influence of the DMSO-d_6_/D_2_O solvent medium.

Native xylan exhibited a resonance near 4.25–4.27 ppm, consistent with the anomeric proton of β-xylopyranosyl residues [6]. Considered alongside the methylation results, this feature supports the structural interpretation of the xylan backbone. Additional ring-region resonances were observed at approximately 3.86, 3.51, 3.25, 3.16, and 3.03 ppm. Weak signals near 5.28 and 5.34 ppm were tentatively associated with arabinose residues, consistent with previous reports on arabinose-containing xylans [36]. These features provide a basis for comparing the native polymer with its sulfated derivatives.

After sulfation, the spectra showed changes in signal position and intensity, including features near 4.98, 4.18, and 3.67 ppm. The redistribution of resonances is consistent with changes in proton environments accompanying chemical modification. Together with the FT-IR results and sulfate assay, these observations support the introduction of sulfate groups into xylan. The downfield features are interpreted collectively as spectral changes associated with sulfation, without assigning them to specific O-2 or O-3 substitution sites. Together with the bulk DS measurements, these spectra support sulfate introduction but do not establish a uniform distribution of sulfate groups along or among xylan chains.

Changes were also observed in the signals near 5.10 and 4.99 ppm and in the weak arabinose-associated region. Because exchangeable-proton intensities depend on solvent composition and proton–deuterium exchange, and overlapping resonances can vary with acquisition conditions, these changes are used for qualitative comparison rather than as direct measures of hydroxyl consumption or side-chain loss. The ^1^H NMR results thus complement the FT-IR and sulfate measurements in characterizing the sulfated derivatives.

### 3.5. FT-IR and UV–Vis Spectrum Analysis

The presence of proteinaceous impurities can be evaluated from characteristic absorption in the UV region, particularly around 280 nm [37]. No pronounced absorption peaks were observed between 260 and 280 nm in the UV–vis spectra of native xylan and its sulfated derivatives (XS1–XS3; Figure S7). These spectra provide a qualitative assessment under the measurement conditions but do not quantify protein or nucleic acid contamination or establish the absence of residual pyridine or other reagents.

**Figure 10:**
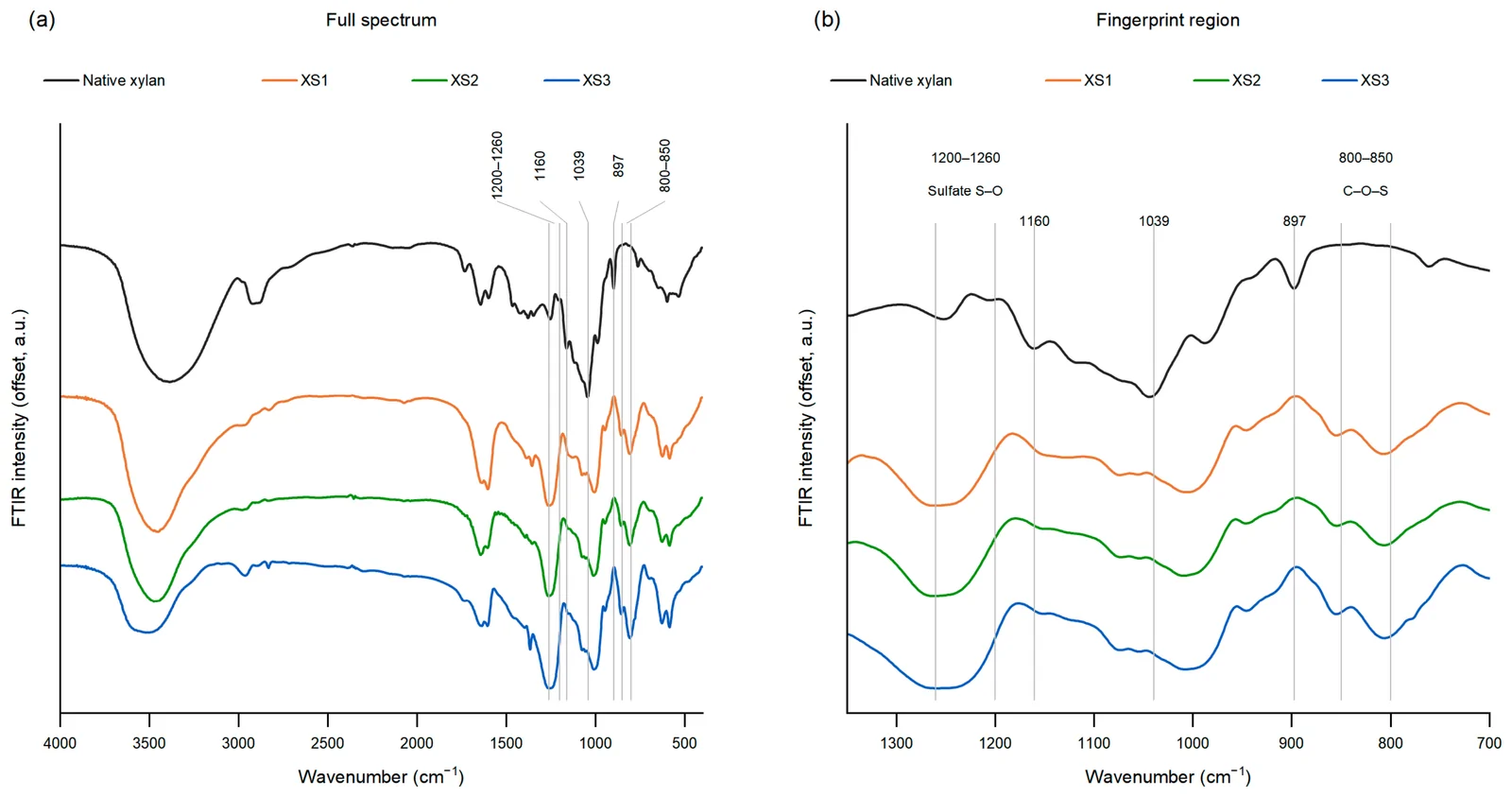
FTIR spectra of native xylan and sulfated derivatives XS1–XS3: (**a**) full spectra over 4000–400 cm^−1^ and (**b**) an enlarged view of the 1350–700 cm^−1^ fingerprint region. Native xylan, XS1, XS2, and XS3 are shown in dark grey, orange, green, and blue, respectively. Annotations mark approximate xylan-associated positions near 1160, 1039, and 897 cm^−1^ and sulfate-associated regions at 1200–1260 cm^−1^ (sulfate-group stretching) and 800–850 cm^−1^ (C–O–S vibration).

Figure 10a compares the FTIR spectra of native xylan and its sulfated derivatives, with the fingerprint region enlarged in Figure 10b. Native xylan showed the characteristic carbohydrate fingerprint, including features near 1160, 1039, and 897 cm^−1^. These regions are associated with glycosidic C–O–C vibrations, overlapping C–O and ring vibrations, and β-linked xylopyranosyl residues, respectively [38,39]. Following sulfation, XS1–XS3 showed more prominent absorption in the 1200–1260 cm^−1^ region and a feature near 810 cm^−1^ within the 800–850 cm^−1^ region, consistent with sulfate-group stretching and C–O–S vibrations [40]. Together with the sulfate assay and NMR results, these spectral changes support the introduction of sulfate ester groups.

### 3.6. I_2_-KI Colorimetric Assay

The iodine-potassium iodide (I_2_-KI) complexation assay serves as a diagnostic structural probe to elucidate polysaccharide branching topologies and screen for starch impurities. Polysaccharides with linear backbones readily intercalate with iodine to form a chromogenic complex, generating a signature absorption maximum at 565 nm. Consequently, the absence of this macroscopic colorimetric response—and its corresponding spectral peak—acts as rigorous confirmation that a polymer is branched and strictly free of starch contamination.

Upon I_2_-KI treatment, both native xylan and its sulfated derivatives lacked any visible chromogenic response, exhibiting absorption maxima localized exclusively at 350 nm with a complete absence of the 565 nm band (Figure S8). This spectral behavior definitively confirms that the native xylan intrinsically possesses a branched architecture. Crucially, the identical spectral profiles of the sulfated derivatives indicate that the sulfation process did not aggressively truncate or compromise this fundamental branching topology. This retained structural heterogeneity corroborates the highly diverse polymeric makeup previously identified via monosaccharide composition profiling. Furthermore, the null chromogenic response definitively excludes residual starch within the extracted fractions, mirroring comparable structural screenings reported by Liu et al. [41].

### 3.7. Polarimetric Analysis

Native xylan exhibited a specific optical rotation of −1.969°, consistent with the predominance of β-configured glycosidic linkages in the xylan backbone and with spectroscopic structural assignments. Similar negative rotations have been reported for xylans from agricultural residues, although their magnitudes vary with botanical origin, branching pattern, and measurement conditions [42].

Experimental optical rotations of polysaccharides may deviate substantially from theoretical random-coil values because of conformational ordering. For example, marine algal xylans exhibit less negative rotations than predicted for ideal random coils, a behavior attributed to extended, ordered β-(1→4)-linked domains [43,44]. Thus, optical rotation can provide complementary information on xylan chain organization and conformation.

Sulfation progressively decreased the magnitude of the specific optical rotation from −1.969° for native xylan to −0.925°, −0.846°, and −0.792° for XS1, XS2, and XS3, respectively. Similar attenuation after sulfation has been reported for other polysaccharides [45]. These changes likely reflect sulfation-induced alterations in the local stereochemical environment and chain conformation, together with the contribution of the introduced sulfate substituents.

### 3.8. Water Solubility Analysis

The water solubility and aggregation behavior of xylan are strongly influenced by its macromolecular structure, including molar mass, branching, monosaccharide composition, and substituent distribution. Sparsely substituted xylans readily form extensive intra- and intermolecular hydrogen bonds, promoting chain association and precipitation, whereas appropriate side-chain substitution enhances hydration and reduces intermolecular aggregation [46]. Native bagasse xylan generally exhibits lower water solubility than more highly substituted xylans, consistent with its relatively low abundance of hydrophilic arabinofuranosyl and glucuronosyl residues [47]. Accordingly, the unmodified bagasse xylan used in this study showed a water solubility of only 21.2 mg/mL.

Residual lignin can further reduce xylan dispersibility by promoting hydrophobic association and may also hinder subsequent chemical modification. Introducing hydrophilic or ionic substituents is therefore an effective strategy to disrupt interchain interactions and improve the water solubility of xylan. Derivatization methods such as sulfation, carboxymethylation, succinylation, and cationization have been widely employed for this purpose, with water solubility often increasing with the degree of substitution (DS).

In this study, the water solubility values of sulfated xylans XS1, XS2, and XS3 increased to 96.8, 115.6, and 130.4 mg/mL, respectively, demonstrating a clear DS-dependent improvement. Sulfation introduces negatively charged sulfate groups onto the xylan backbone, increasing electrostatic repulsion and steric hindrance while weakening interchain hydrogen bonding. These effects enhance chain hydration and dispersion in water and reduce intermolecular association [48]. The increase in water solubility may contribute to the functional properties of the sulfated derivatives.

### 3.9. Macromolecular and Colloidal Characterization

#### 3.9.1. Concentration-Dependent Apparent DLS Responses and ζ-Potential

**Figure 11:**
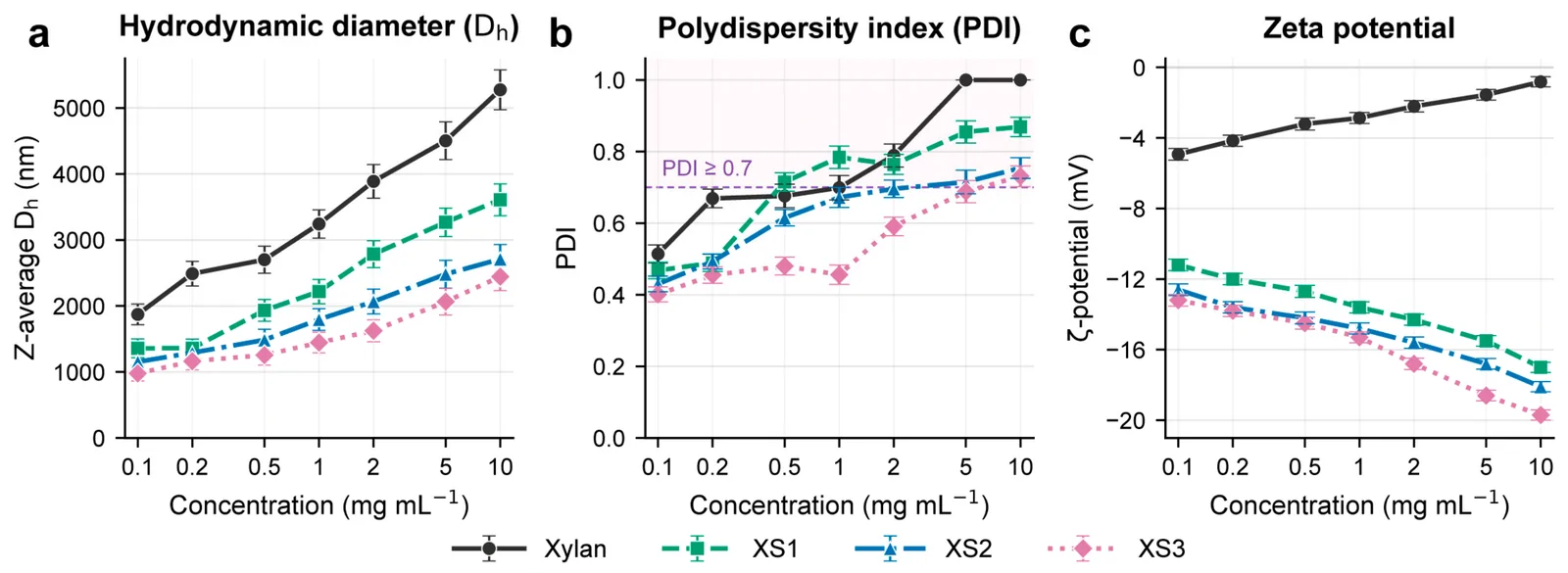
Concentration-dependent properties of native xylan and sulfated xylan derivatives (XS1–XS3): (**a**) DLS-derived apparent hydrodynamic diameter (Z-average); (**b**) polydispersity index (PDI); and (**c**) ζ-potential. The DLS diameters represent instrument-reported apparent sizes of the scattering populations, rather than the actual size of individual polymer chains.”.

As shown in Figure 11a, the instrument-reported Z-average hydrodynamic diameters of native xylan, XS1, XS2, and XS3 at 100 μg/mL were (1.9 ± 0.2) × 10^3^, (1.4 ± 0.1) × 10^3^, (1.2 ± 0.1) × 10^3^, and (1.0 ± 0.1) × 10^3^ nm, respectively. At 10 mg/mL, the corresponding values increased to (5.3 ± 0.3) × 10^3^, (3.6 ± 0.2) × 10^3^, (2.7 ± 0.2) × 10^3^, and (2.4 ± 0.2) × 10^3^ nm. The latter outputs are retained as descriptive instrumental observations and are not used to infer quantitative aggregate dimensions. The sulfated derivatives consistently showed lower apparent diameters than native xylan at the same concentration.

The PDI of native xylan increased from 0.51 ± 0.03 at 100 μg/mL to 1.000 (SD = 0) at 10 mg/mL (Figure 11b). Over the same concentration range, the values for XS1, XS2, and XS3 increased from 0.47 ± 0.02, 0.43 ± 0.02, and 0.40 ± 0.02 to 0.87 ± 0.03, 0.75 ± 0.03, and 0.73 ± 0.03, respectively. These values indicate broad, heterogeneous scattering populations. Although the sulfated derivatives had lower PDI values than native xylan, all samples remained highly polydisperse, particularly at 10 mg/mL.

The apparent ζ-potential of native xylan shifted from −4.9 ± 0.3 to −0.8 ± 0.3 mV as the concentration increased from 100 μg/mL to 10 mg/mL (Figure 11c). For XS1, XS2, and XS3, the corresponding values changed from −11.2 ± 0.4, −12.6 ± 0.4, and −13.2 ± 0.3 mV to −17.0 ± 0.3, −18.1 ± 0.3, and −19.7 ± 0.3 mV, respectively. The more negative values of the sulfated derivatives are consistent with the introduction of anionic sulfate groups.

#### 3.9.2. Relationship Between Chromatographic Molar Mass and Aqueous Association

The combined D_h_, PDI, and ζ-potential results suggest that increasing concentration favors aqueous association, whereas sulfation may limit this association through electrostatic and hydration effects (Figure S9). Higher concentrations generally produced larger apparent DLS diameters and higher PDI values. At corresponding concentrations, XS1–XS3 showed lower apparent diameters and PDI values and more negative ζ-potentials than native xylan. These trends are consistent with reduced large-scale association after sulfation. The introduction of anionic sulfate groups may increase electrostatic repulsion and hydration, although the contributions of these effects were not measured separately. Related concentration- and substitution-dependent association behavior has been reported for xylan and other sulfated polysaccharides [49,50,51,52].

The HPGPC and DLS results describe different physical properties and sample populations. Dextran-calibrated HPGPC yielded apparent molar masses (M_w,app_) of 5.56, 5.79, 5.92, and 6.30 kDa for native xylan, XS1, XS2, and XS3, respectively. These estimates apply to the fraction that dissolved in the mobile phase, passed through sample filtration, and eluted through the SEC columns; they do not establish absolute single-chain molar masses. DLS, in contrast, measures diffusion-derived apparent hydrodynamic sizes of scattering species in aqueous dispersions. Even a small population of large assemblies can contribute disproportionately to the signal. The increase in M_w,app_ after sulfation can therefore coexist with lower apparent DLS diameters because the two methods characterize different properties under different solution conditions. HPGPC retention reflects the chromatographically accessible fraction in the electrolyte-containing mobile phase, whereas the aqueous DLS signal may be dominated by larger assemblies.

Even at 100 μg/mL, the approximately 1–2 μm DLS diameters are compatible with large supramolecular assemblies rather than isolated xylan chains. At 10 mg/mL, the larger reported diameters and high PDI indicate broad scattering populations for which quantitative size interpretation is limited. Possible sedimentation, nonideal scattering, and differences between the assumed and actual dispersion viscosity may further affect these estimates. We therefore interpret the concentration-dependent DLS trends qualitatively as changes in aqueous association. Without independent microscopy and systematic dilution measurements, the data cannot establish aggregate abundance, morphology, internal structure, or absolute dimensions, and do not justify assigning the scattering species to micelles, nanoparticles, or other defined architectures. Within these limits, the results suggest that sulfation reduces large-scale association without eliminating it.

### 3.10. Rheological Profiling

#### 3.10.1. Steady-State Flow Dynamics

**Figure 12:**
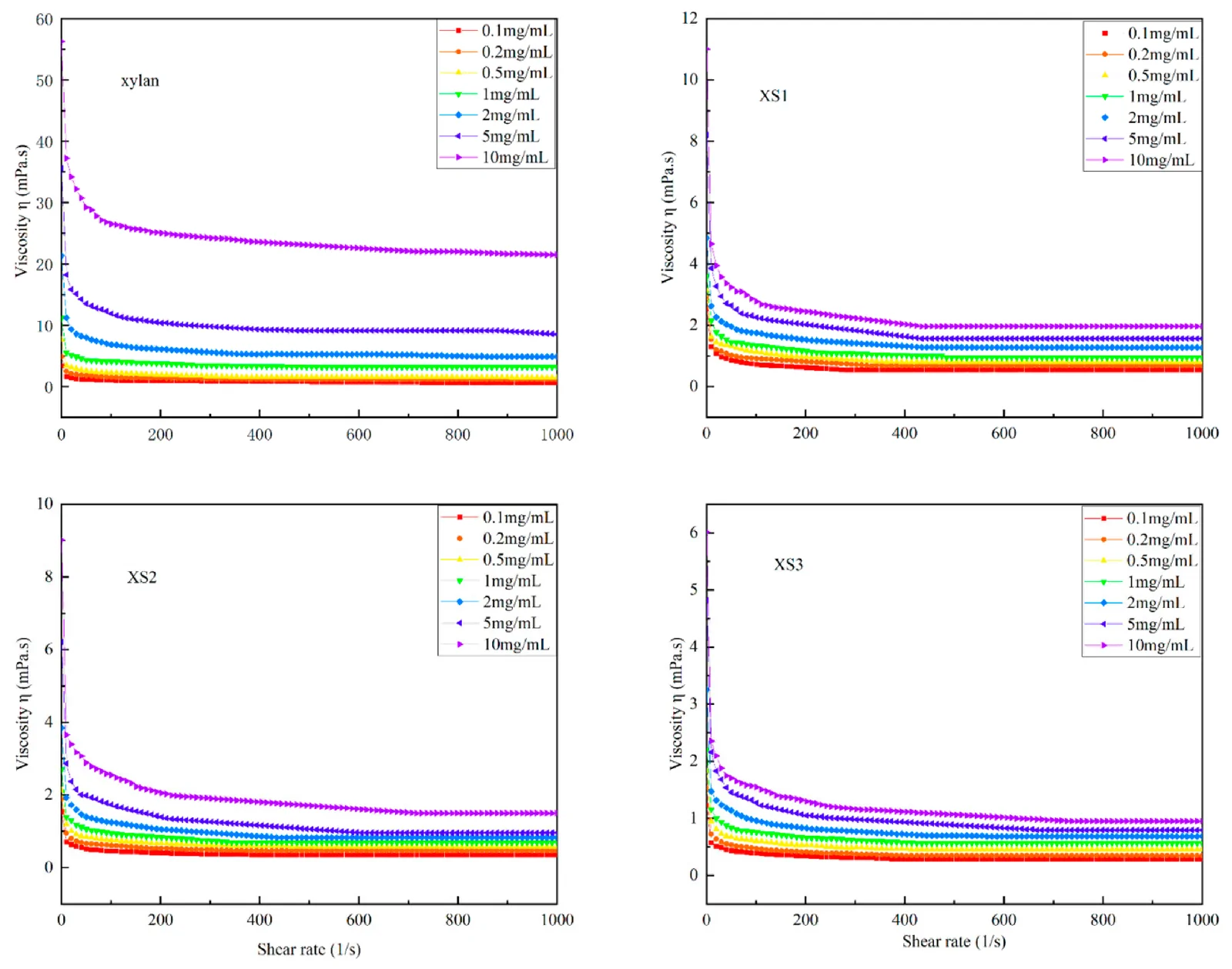
Shear rate dependence of apparent viscosity for native xylan (A) and its sulfated derivatives (XS1–XS3) across varying concentrations.

Both native xylan and its sulfated derivatives exhibited typical non-Newtonian shear-thinning behavior, with apparent viscosity decreasing continuously as shear rate increased (Figure 12). This behavior can be attributed to shear-induced chain orientation, disentanglement, and disruption of transient intermolecular associations. At low shear rates, reversible hydrogen bonding and chain interactions maintain a transient network and relatively high viscosity, whereas increasing shear progressively weakens these interactions and promotes chain alignment, consistent with the dynamic-network interpretation of polysaccharide flow behavior [53].

Sulfation markedly reduced the apparent viscosity of xylan. The introduction of negatively charged sulfate groups increases electrostatic repulsion and steric separation between neighboring chains, thereby weakening intermolecular association and reducing flow resistance. Similar rheological changes have been reported for other sulfated polysaccharides, including β-glucans and pectin [54,55]. In contrast, increasing polymer concentration increased viscosity because greater chain proximity promotes intermolecular interactions and hydrodynamic overlap, whereas dilute solutions displayed weaker interactions and more nearly Newtonian behavior.

Solution rheology can also be influenced by ionic strength through electrostatic screening. Although the sulfated xylans were present as sodium salts, no additional electrolyte was introduced; therefore, external ionic screening was considered limited. The observed viscosity may reflect contributions from chain dimensions, flexibility, charge, and intermolecular association.

The steady-state flow curves were fitted using the Ostwald–de Waele power-law model:

(5) $$\eta \;=\;K\times {\left(\gamma ˙\right)}^{n-1}$$

where *η* is the apparent viscosity (mPa s), γ̇ is the shear rate (s^−1^), *K* is the consistency index (mPa s^n^), and *n* is the dimensionless flow behavior index. All samples exhibited *n* < 1 (Tables S5–S8), confirming pseudoplastic behavior. Native xylan generally showed higher K values than the sulfated derivatives, indicating stronger intermolecular interactions and greater resistance to flow.

The concentration dependence of K was further evaluated using log K–log C relationships. The relatively low scaling slopes (0.311–0.643; Tables S5–S8) indicate a weak concentration dependence of rheological consistency over the tested range, suggesting limited development of an extended entangled network. These results are consistent with the low molecular weight and relatively weak intermolecular association of both native and sulfated xylans.

#### 3.10.2. Dynamic Frequency Sweep Analysis

**Figure 13:**
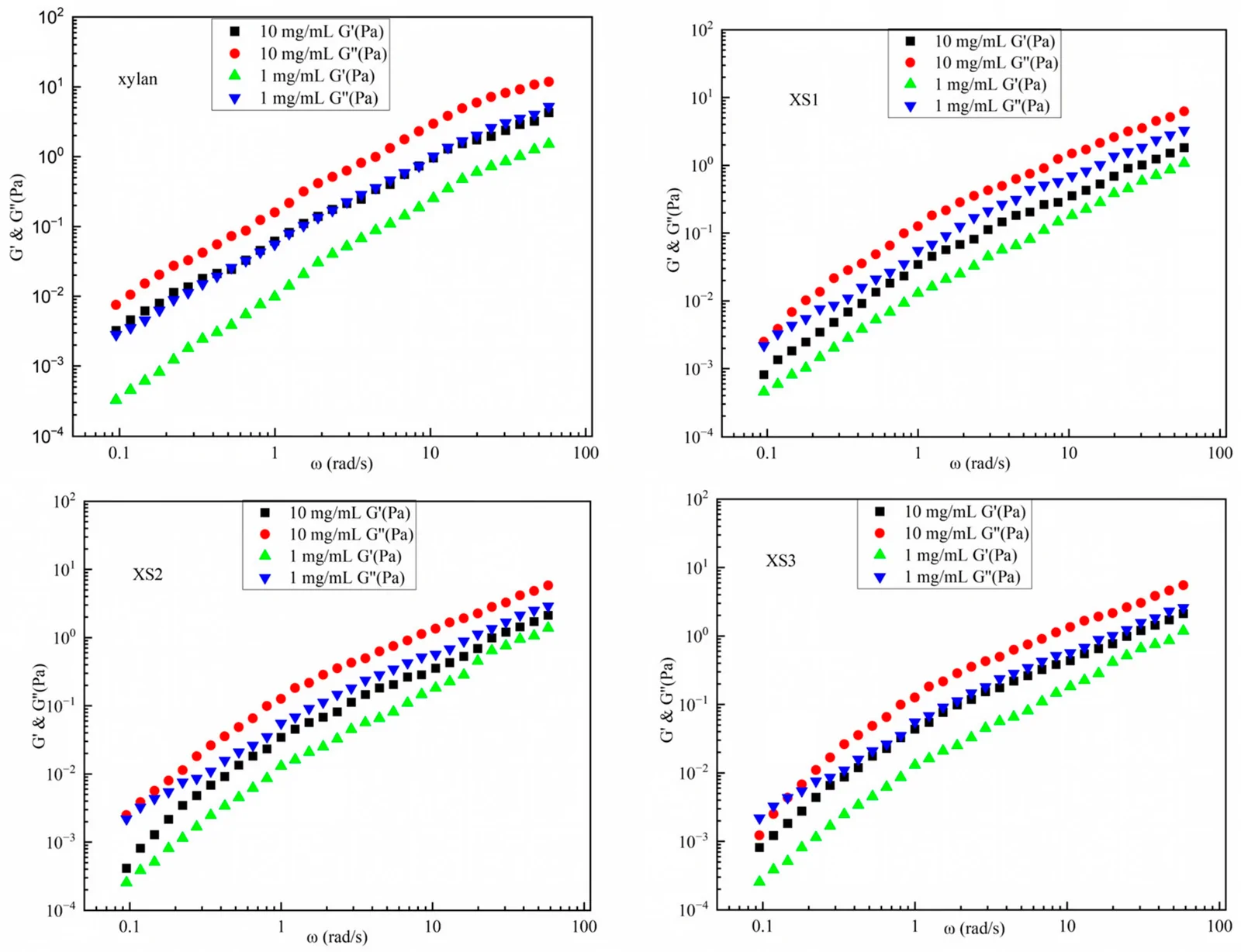
Dynamic rheological profiles of native xylan and its sulfated derivatives (XS1–XS3). Frequency dependence of the storage (*G′*) and loss (*G″*) moduli across varying polymer concentrations.

Small-amplitude oscillatory shear (SAOS) measurements were used to characterize the viscoelastic behavior of native and sulfated xylans. Over the investigated frequency range (0.1–100 rad/s), the loss modulus (*G″*) remained higher than the storage modulus (*G′*), with tan *δ* > 1 and no *G′*–*G″* crossover (Figure 13). These results indicate predominantly viscous, liquid-like behavior rather than formation of a continuous gel network.

Both *G′* and *G″* increased with polymer concentration, reflecting enhanced intermolecular interactions and transient chain overlap at higher concentrations. In contrast, sulfation reduced both moduli relative to native xylan. The introduction of sulfate groups weakens intermolecular hydrogen bonding and increases electrostatic repulsion and hydration, thereby reducing chain association and network strength [56,57].

The frequency dependence of *G′* and *G″* was fitted using the power-law relationships *G′* = *k′ω^n^′* and *G″* = *k″ω^n^″*, where *k′* and *k″* are consistency coefficients and *n′* and *n″* are frequency-scaling exponents. The fitted parameters (Table 3) confirmed frequency-dependent viscoelastic behavior, while the persistent dominance of *G″* over *G′* further supported the liquid-like character of all samples.

Overall, sulfation weakened intermolecular associations and shifted xylan toward a more hydrated and electrostatically stabilized polyelectrolyte system. This rheological modification provides useful structure–property information for tailoring the processing and formulation behavior of xylan-based materials.

### 3.11. In Vitro Coagulation-Time Responses

#### 3.11.1. Response of Native Xylan in the Coagulation Assays

The activated partial thromboplastin time (APTT), prothrombin time (PT), and thrombin time (TT) serve as standard clinical diagnostic indices to evaluate the intrinsic, extrinsic, and common coagulation cascades (fibrinogen-to-fibrin conversion), respectively. Pharmacologically, a compound is classified as an active anticoagulant if it prolongs PT and TT by >3 s and APTT by >10 s relative to normal baseline values, with greater temporal prolongations reflecting superior inhibitory potency. Across the tested concentration range, native xylan did not measurably prolong APTT, PT, or TT relative to the physiological-saline control. Thus, no detectable anticoagulant effect of native xylan was observed under the present assay conditions.

#### 3.11.2. Coagulation-Time Responses of Sulfated Xylans

The anticoagulant activity of sulfated xylans is well established [5,58,59]. Here, XS1–XS3 were compared under the same plasma-assay conditions to determine how their measured coagulation-time responses varied across the selected derivative series.

**Figure 14:**
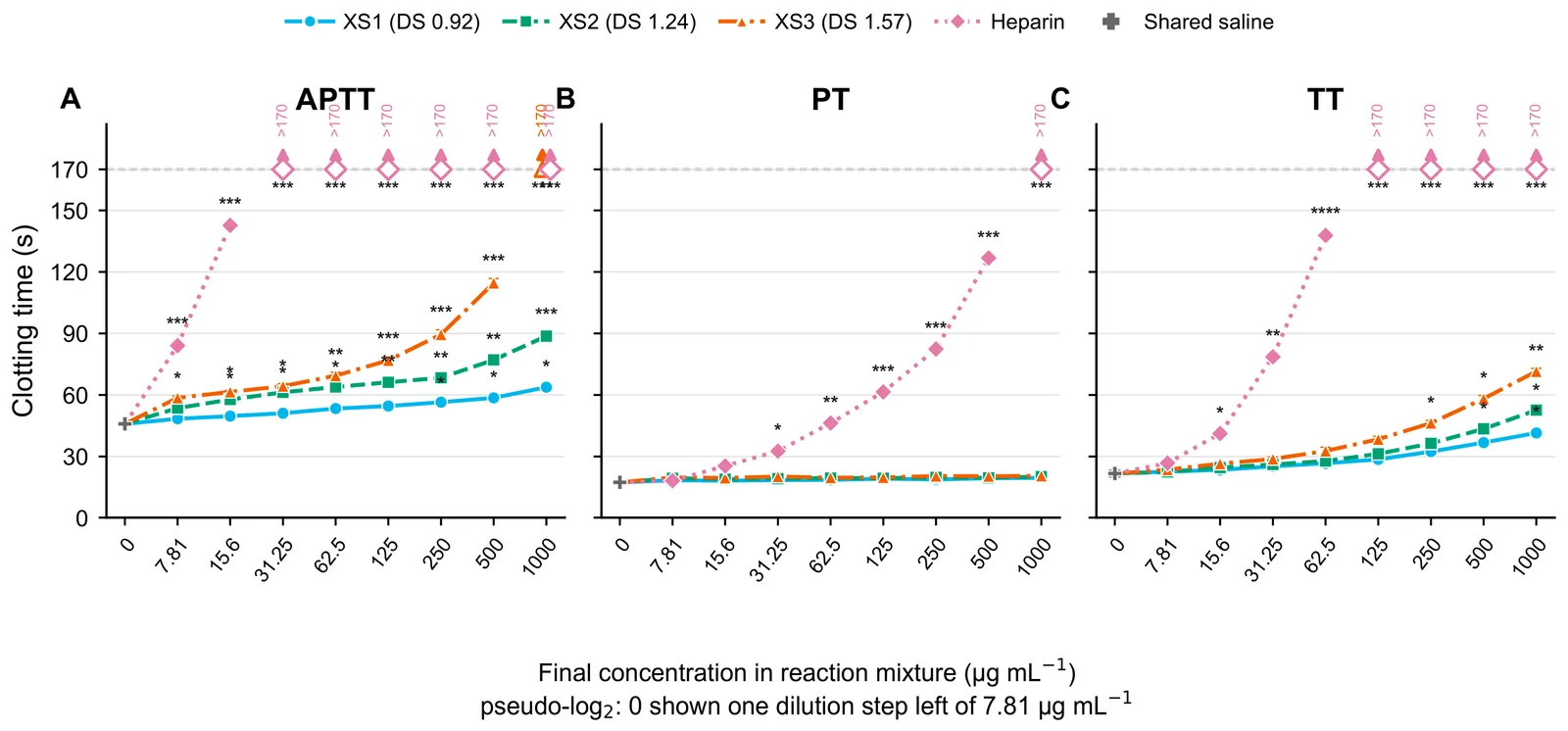
Concentration–response profiles of the in vitro anticoagulant activity of xylan sulfates (XS1–XS3): (**A**) acti-vated partial thromboplastin time (APTT); (**B**) prothrombin time (PT); and (**C**) thrombin time (TT). Heparin served as the positive control and saline as the negative control. * *p* < 0.05, ** *p* < 0.01, *** *p* < 0.001, and **** *p* < 0.0001 versus the saline control.

XS1–XS3 prolonged APTT and TT in a concentration-dependent manner over the tested range (Figure 14). At a final concentration of 1000 μg mL^−1^ in the reaction mixture, APTT values were approximately 64 ± 2, 89 ± 2, and >170 s for XS1, XS2, and XS3, respectively, compared with 46 ± 1 s for the saline control. The corresponding TT values were 42 ± 2, 53 ± 1, and 72 ± 2 s, versus 22 ± 2 s for saline. PT showed smaller changes, with values of 19.6 ± 0.5, 20.2 ± 0.5, and 20.6 ± 0.6 s, respectively, compared with 17.4 ± 0.8 s. Heparin produced clotting times >170 s at the tested concentrations of ≥31.25 μg mL^−1^ for APTT, ≥125 μg mL^−1^ for TT, and 1000 μg mL^−1^ for PT. Values reported as >170 s are right-censored observations and do not permit quantitative comparisons beyond that threshold.

The greater prolongation of APTT and TT relative to PT resembles profiles reported for other sulfated polysaccharides, including sodium alginate sulfates and quaternary ammonium chitosan sulfates [60,61]. Previous studies have also shown that anticoagulant responses depend on properties other than sulfate content. Suwan et al. reported molecular-weight-dependent activity mediated mainly through heparin cofactor II (HC-II) for their sulfated chitosan preparations [62], while Zhang et al. found that reducing molecular size substantially decreased the activity of smaller fragments derived from a sulfated polysaccharide from *Monostroma latissimum* [63]. These findings support considering molar mass alongside sulfate substitution when interpreting the present sample series. Across XS1–XS3, the extent of APTT and TT prolongation increased as DS rose from 0.92 to 1.57, dextran-equivalent apparent molar mass increased from approximately 5.79 to 6.30 kDa, and the measured ζ-potentials became more negative. Given the concurrent variations in these physicochemical properties, the observed anticoagulant activity cannot be attributed exclusively to sulfate group density, molar mass, or electrostatic characteristics.

## 4. Conclusions

The conversion of abundant lignocellulosic biomass into high-value biomedical materials is fundamentally bottlenecked by the steric constraints of native biopolymers and the destructive nature of traditional functionalization methods. Sugarcane-bagasse xylan was sulfated using SO_3_·Py in a formulation containing DMAP and EDCI. Single-factor experiments described the changes in DS and DS-adjusted isolated yield with reagent loading, temperature and time, while an entropy-weighted five-factor Box–Behnken design identified a compromise between substitution and product recovery. The model-predicted compromise optimum (DMAP/xylan = 0.218, SO_3_·Py/xylan = 3.246, EDCI/xylan = 2.198, 50.73 °C, and 3.79 h) gave a predicted composite score of 0.782. Three independent validation batches produced DS = 1.53 ± 0.01, an isolated yield of 78.0 ± 0.1%, and a composite score of 0.792 ± 0.008, demonstrating good local reproducibility of the optimized process.

The strategic covalent grafting of anionic sulfate half-esters triggers profound topological and thermodynamic transitions within the biopolymer network. Comprehensive physicochemical, colloidal, and rheological profiling confirms that sulfation effectively dismantles the native interchain hydrogen-bonding networks. This structural reconfiguration transitions the polymer from a poorly water solubility, highly aggregated random coil into an extended, highly water solubility, and electrostatically stable polyelectrolyte, simultaneously unlocking highly tunable rheological dynamics. Across the XS1–XS3 series (DS = 0.92, 1.24, and 1.57), sulfation increased water solubility from 21.2 mg/mL for native xylan to 96.8–130.4 mg/mL and was accompanied by more negative ζ-potentials, smaller apparent hydrodynamic aggregates, and altered steady-shear and dynamic rheological responses. Together, these observations are consistent with increased hydration and electrostatic repulsion after sulfate introduction. Because the HPGPC values are dextran-equivalent apparent molar masses and the DLS measurements reflect hydrated supramolecular assemblies. These complementary measurements therefore indicate sulfation-associated changes in aqueous organization rather than a direct inverse relationship between absolute single-chain molecular mass and aggregate size.

Crucially, this engineered structural reconfiguration strictly dictates biological function. By replicating the dense negative-charge microenvironment characteristic of mammalian heparin, the optimized sulfated xylans exhibited measurable in vitro anticoagulant activity. In vitro, XS1–XS3 prolonged APTT and TT in a concentration-dependent manner, whereas PT showed smaller changes under the tested conditions; native xylan showed no detectable effect under the tested conditions. The increasing anticoagulant response across XS1–XS3 paralleled increasing DS, but DS also covaried with apparent molar mass and potentially other structural features. Therefore, the present data support a structure–activity association rather than a causal assignment to sulfate density alone. Overall, sugarcane-bagasse xylan is a tunable precursor for water-soluble sulfated polysaccharides with measurable in vitro anticoagulant activity.

## Figures and Tables

**Table 1:** Glycosidic linkage analysis of bagasse xylan determined via methylation.

Retention Time (min)	Mass Fragments (*m*/*z*)	Type of Linkage	Molar Ratio
8.18	43, 59, 71, 87, 102, 118, 129, 145, 161	Araf-(1→	0.40
8.599	43, 59, 73, 88, 101, 117, 129, 162	Xyl*p*-(1→	0.19
9.237	43, 71, 101, 118, 129, 161, 190, 233	→2)-Araf-(1→	0.06
9.592	43, 87, 102, 118, 129, 189, 234	→3,6)-Gal*p*-(1→	0.07
9.68	43, 59, 71, 87, 102, 118, 129, 162, 189	→4)-Xyl*p*-(1→	5.03
10.551	43, 59, 74, 85, 99, 118, 129, 160, 201, 261	→3,4)-Xyl*p*-(1→	0.79
11.136	43, 57, 71, 87, 99, 118, 129, 173, 233	→4)-Glc*p*-(1→	0.09

Note: Araf, arabinofuranosyl residues; Xyl*p*, xylopyranosyl residues; Gal*p*, galactopyranosyl residues; Glc*p*, glucopyranosyl residues; *m*/*z*, mass-to-charge ratio.

**Table 2:** Verification of experimental results.

No	DS	Y/%	S_i_
1	1.53	78.12	0.79448
2	1.54	77.93	0.79874
3	1.52	78.04	0.78382
$\bar{x}$	1.53	78.0	0.792
SD	0.01	0.1	0.008
RSD%	0.65%	0.1%	0.970%

**Table 3:** Rheological power-law scaling parameters for the viscoelastic moduli (*G′* and *G″*) of native and sulfated xylans.

Sample	Concentration (mg/mL)	*G′* = *k’(ω)^n^′*			*G″* = *k″(ω)^n^″*		
		*k′*	*n′*	R^2^	*k″*	*n″*	R^2^
Xylan	1	0.0022	1.4065	0.99	0.0136	1.2725	0.99
	10	0.0194	1.1456	0.98	0.0923	1.0503	0.99
XS1	1	0.0009	1.5249	0.99	0.0113	1.2153	0.98
	10	0.0044	1.2933	0.98	0.0340	1.1117	0.98
XS2	1	0.0005	1.6902	0.99	0.0077	1.2774	0.98
	10	0.0020	1.5010	0.98	0.0298	1.1240	0.98
XS3	1	0.0009	1.5452	0.99	0.0116	1.1590	0.98
	10	0.0072	1.2126	0.98	0.0356	1.0708	0.98

## Data Availability

The original contributions presented in this study are included in the article/Supplementary Materials. Further inquiries can be directed to the corresponding authors.

## Supplementary Materials

The following supporting information can be downloaded at https://www.mdpi.com/article/10.3390/polym18182296/s1. Table S1: Chemical composition of the xylan; Table S2: Independent process variables and their coded experimental levels for the Box-Behnken design; Table S3: Experimental design matrix and corresponding empirical responses for the Box-Behnken response surface methodology; Table S4: Analysis of variance (ANOVA) of the composite scoring model for xylan sulfate synthesis utilizing a three-level Box-Behnken design; Table S5: Rheological power-law modeling parameters for the apparent viscosity of native xylan; Table S6: Rheological power-law modeling parameters for the apparent viscosity of xylan sulfate (XS1); Table S7: Rheological power-law modeling parameters for the apparent viscosity of xylan sulfate (XS2); Table S8: Rheological power-law modeling parameters for the apparent viscosity of xylan sulfate (XS3); Figure S1: The methylation treatment process of xylan; Figure S2: Hypothetical involvement of DMAP in xylan sulfation. The proposed intermediates and regeneration steps illustrate a working hypothesis; Figure S3: Hypothetical EDCI-mediated sulfate-transfer pathway; Figure S4: Model effects, global process-factor sensitivity, and internal predictive validation of the entropy-weighted quadratic response-surface model for sugarcane-bagasse xylan sulfation; Figure S5: Two-factor interactions governing the model-predicted composite score for the sulfation of sugarcane-bagasse xylan; Figure S6: Response-specific decomposition of the optimization landscape reveals an interior compromise between xylan sulfation and product recovery; Figure S7: UV–vis spectra of native xylan and its sulfated derivatives (XS1–XS3), measured at 2 mg/mL in ultrapure water over 200–400 nm, with ultrapure water as the reference; Figure S8: UV-Vis absorption spectra of native and sulfated xylans following I2-KI complexation; Figure S9: Proposed structure–property cascade of xylan sulfation.
