# Supplementary material for: Sulfation of Sugarcane-Bagasse Xylan: Process Optimization, Physicochemical Properties and In Vitro Anticoagulant Activity
Source: Polymers (Basel). 2026 Sep 19;18(18):2296. doi: 10.3390/polym18182296 (PMC13611467; doi:10.3390/polym18182296)
Supplement: Supplementary file 1 [file polymers-18-02296-s001.zip › polymers-4559342-supplementary.pdf]

Supplementary Information

Table S1. Chemical composition of the xylan

No.	Total carbohydrate(%)	Proteins (%)	Phenolic Compounds (%)	Molar Ratio* (%)					Mw,app (Da)
				Xyl	Glu	Gal	Ara	GlcA	
X-31000	95.3	n.d.	n.d.	77.01	5.87	2.68	13.79	0.66	5,559

Note: Total carbohydrate, protein, and phenolic contents were determined using the phenol–sulfuric acid, Bradford, and Folin–Ciocalteu assays, respectively, with D-glucose, bovine serum albumin (BSA), and gallic acid as the corresponding calibration standards.

*Molar ratio obtained by High-Performance Anion-Exchange Chromatography (HPAEC) analyses after acid hydrolysis (TFA 2 M; 2 h; 121 °C). Xyl: xylose; Glu: glucose; Gal: galactose; Ara: arabinose; GlcA: Glucuronic acid.

The apparent weight-average molar mass (Mw,app) of xylan was determined by high-performance gel permeation chromatography (HPGPC).

n.d.: Not detectable in the evaluated conditions.

Table S2. Independent process variables and their coded experimental levels for the Box-Behnken design.

Factors	Parameters	Units	Levels		
			-1	0	1
A	DMAP/xylan mass ratio	g/g	0.15	0.20	0.25
B	SO_3·Py/xylan mass ratio	g/g	1	3	5
C	EDCI/xylan mass ratio	g/g	1	2	3
D	reaction temperature	°C	40	50	60
E	reaction time	h	2	4	6

Note: The levels listed here apply to the Box–Behnken design. Fixed conditions for the preliminary single-factor series are specified separately in the captions of Figs. 1–5. A, B and C denote reagent-to-xylan mass ratios (g/g) for DMAP, SO_3·Py and EDCI, respectively. The SO_3·Py mass includes the complete sulfur trioxide–pyridine complex; EDCI refers to the hydrochloride reagent. D and E denote reaction temperature (°C) and reaction time (h). The coded level 0 represents the design centre.

Table S3. Experimental design matrix and corresponding empirical responses for the Box-Behnken response surface methodology.

Run	A	B	C	D	E	DS	Y/%	S_i
1	0	0	0	0	0	1.47	78.83	0.76
2	0	0	1	0	1	1.51	71.87	0.63
3	1	1	0	0	0	1.53	72.21	0.65
4	0	0	0	0	0	1.44	78.45	0.72
5	0	0	1	1	0	1.41	71.38	0.53
6	0	1	0	1	0	1.42	72.13	0.56
7	0	-1	0	-1	0	1.07	80.13	0.44

8	0	0	-1	0	-1	1.18	81.28	0.56
9	1	0	0	0	-1	1.36	78.53	0.66
10	0	1	0	-1	0	1.41	67.14	0.43
11	-1	-1	0	0	0	0.96	83.41	0.42
12	-1	1	0	0	0	1.12	77.83	0.43
13	0	-1	0	0	1	1.21	76.21	0.47
14	0	0	0	-1	-1	1.22	76.36	0.48
15	-1	0	0	-1	0	1.08	76.36	0.36
16	0	0	0	1	1	1.37	71.37	0.49
17	-1	0	1	0	0	1.23	78.23	0.53
18	1	-1	0	0	0	1.27	76.27	0.52
19	-1	0	0	0	1	1.14	79.25	0.48
20	0	-1	1	0	0	1.29	75.14	0.51
21	0	0	0	1	-1	1.34	76.37	0.59
22	1	0	0	-1	0	1.29	76.12	0.54
23	0	-1	-1	0	0	1.07	79.26	0.42
24	0	0	1	0	-1	1.47	74.26	0.65
25	-1	0	-1	0	0	0.92	85.32	0.43
26	1	0	-1	0	0	1.34	76.82	0.60
27	0	1	0	0	1	1.42	72.25	0.56
28	0	-1	0	0	-1	1.19	79.82	0.54
29	-1	0	0	1	0	1.09	79.24	0.44
30	0	-1	0	1	0	1.13	76.14	0.40
31	0	1	-1	0	0	1.22	76.19	0.48
32	1	0	0	1	0	1.45	71.76	0.57
33	-1	0	0	0	-1	1.18	78.83	0.50
34	0	0	0	-1	1	1.21	76.65	0.48
35	0	0	-1	1	0	1.23	76.18	0.49
36	0	0	1	-1	0	1.31	75.38	0.54
37	0	1	0	0	-1	1.33	73.52	0.51
38	0	0	0	0	0	1.52	78.34	0.79
39	0	0	0	0	0	1.43	79.55	0.74
40	1	0	1	0	0	1.57	71.51	0.67
41	0	0	-1	0	1	1.24	77.66	0.53
42	0	0	-1	-1	0	1.06	78.17	0.38
43	0	1	1	0	0	1.43	71.35	0.55
44	1	0	0	0	1	1.44	72.68	0.59
45	0	0	0	0	0	1.53	77.12	0.77
46	0	0	0	0	0	1.49	78.83	0.78

Note: XS1, XS2, and XS3 correspond to Runs 25, 41, and 40, respectively, and were selected for subsequent characterization on the basis of their measured DS values. Selection was not based on yield maximization. A–E are coded factor levels; the corresponding actual values and units are given in Table S2.Y (%) denotes DS-adjusted isolated yield calculated using Eq. (2) in the main text.

Table S4. Analysis of variance (ANOVA) of the composite scoring model for xylan sulfate synthesis utilizing a three-level Box-Behnken design.

Source	Sum of Squares	df	Mean Square	F-value	*p*-value	
Model	0.54752	20	0.02738	60.62400	< 0.0001	significant
A	0.09135	1	0.09135	202.30000	< 0.0001	
B	0.01236	1	0.01236	27.37800	< 0.0001	
C	0.03319	1	0.03319	73.49300	< 0.0001	
D	0.01073	1	0.01073	23.75300	< 0.0001	
E	0.00427	1	0.00427	9.45240	0.00505	
AB	0.00382	1	0.00382	8.45930	0.00751	
AC	0.00020	1	0.00020	0.43264	0.51671	
AD	0.00040	1	0.00040	0.88502	0.35583	
AE	0.00047	1	0.00047	1.03810	0.31801	
BC	0.00017	1	0.00017	0.36878	0.54914	
BD	0.00712	1	0.00712	15.77500	0.00053	
BE	0.00341	1	0.00341	7.54490	0.01100	
CD	0.00297	1	0.00297	6.56870	0.01678	
CE	0.00003	1	0.00003	0.07446	0.78719	
DE	0.00203	1	0.00203	4.50420	0.04390	
A^2	0.10520	1	0.10520	232.97000	< 0.0001	
B^2	0.20309	1	0.20309	449.74000	< 0.0001	
C^2	0.08886	1	0.08886	196.78000	< 0.0001	
D^2	0.24371	1	0.24371	539.69000	< 0.0001	
E^2	0.05999	1	0.05999	132.84000	< 0.0001	
Residual	0.01129	25	0.00045			
Lack of Fit	0.00824	20	0.00041	0.67606	0.75929	not significant
Pure Error	0.00305	5	0.0006095			
Cor Total	0.55881	45				

Table S5 Rheological power-law modeling parameters for the apparent viscosity of native xylan.

Concentration（mg/mL）	Ostwald-de Waele Model	K	n	R2	Log K-Log C	R2
0.1	$\eta = 2.299\times(\dot{\gamma})-0.177$	2.299	0.823	0.986		
0.2	$\eta = 3.341\times(\dot{\gamma})-0.162$	3.341	0.838	0.986		
0.5	$\eta = 5.055\times(\dot{\gamma})-0.181$	5.055	0.819	0.988		
1	$\eta = 7.898\times(\dot{\gamma})-0.140$	7.898	0.860	0.989	Log K =0.643 Log C+0.956	0.99
2	$\eta = 14.678\times(\dot{\gamma})-0.163$	14.678	0.837	0.991		
5	$\eta = 24.658\times(\dot{\gamma})-0.156$	24.658	0.844	0.988		
10	$\eta = 44.412\times(\dot{\gamma})-0.106$	44.412	0.894	0.990		

Table S6 Rheological power-law modeling parameters for the apparent viscosity of xylan sulfate (XS1).

Concentration（mg/mL）	Ostwald-de Waele Model	K	n	R^2	Log K-Log C	R^2
0.1	$\eta = 1.770\times(\dot{\gamma})^{-0.186}$	1.770	0.814	0.971		
0.2	$\eta = 2.068\times(\dot{\gamma})^{-0.174}$	2.068	0.826	0.980		
0.5	$\eta = 2.310\times(\dot{\gamma})^{-0.162}$	2.310	0.838	0.979		
1	$\eta = 2.717\times(\dot{\gamma})^{-0.159}$	2.717	0.841	0.982	Log K =0.311 Log C+0.505	0.95
2	$\eta = 3.587\times(\dot{\gamma})^{-0.159}$	3.587	0.841	0.988		
5	$\eta = 5.657\times(\dot{\gamma})^{-0.197}$	5.657	0.803	0.991		
10	$\eta = 7.350\times(\dot{\gamma})^{-0.205}$	7.350	0.795	0.991		

Table S7. Rheological power-law modeling parameters for the apparent viscosity of xylan sulfate (XS2).

Concentration（mg/mL）	Ostwald-de Waele Model	K	n	R^2	Log K-Log C	R^2
0.1	$\eta = 1.069\times(\dot{\gamma})^{-0.175}$	1.069	0.825	0.977		
0.2	$\eta = 1.344\times(\dot{\gamma})^{-0.167}$	1.344	0.833	0.976		
0.5	$\eta = 1.567\times(\dot{\gamma})^{-0.155}$	1.567	0.845	0.982		
1	$\eta = 1.980\times(\dot{\gamma})^{-0.162}$	1.980	0.838	0.980	Log K =0.375 Log C+0.358	0.97
2	$\eta = 2.770\times(\dot{\gamma})^{-0.183}$	2.770	0.817	0.984		
5	$\eta = 4.247\times(\dot{\gamma})^{-0.215}$	4.247	0.785	0.982		
10	$\eta = 6.142\times(\dot{\gamma})^{-0.205}$	6.142	0.795	0.994		

Table S8. Rheological power-law modeling parameters for the apparent viscosity of xylan sulfate (XS3).

Concentration（mg/mL）	Ostwald-de Waele Model	K	n	R^2	Log K-Log C	R^2
0.1	$\eta = 0.871\times(\dot{\gamma})^{-0.174}$	0.871	0.826	0.988		
0.2	$\eta = 1.076\times(\dot{\gamma})^{-0.177}$	1.076	0.823	0.987		
0.5	$\eta = 1.308\times(\dot{\gamma})^{-0.165}$	1.308	0.835	0.979		
1	$\eta = 1.597\times(\dot{\gamma})^{-0.161}$	1.597	0.839	0.984	Log K =0.338 Log C+0.251	0.99
2	$\eta = 2.266\times(\dot{\gamma})^{-0.185}$	2.266	0.815	0.992		
5	$\eta = 3.262\times(\dot{\gamma})^{-0.209}$	3.262	0.791	0.996		
10	$\eta = 3.977\times(\dot{\gamma})^{-0.213}$	3.977	0.787	0.999		

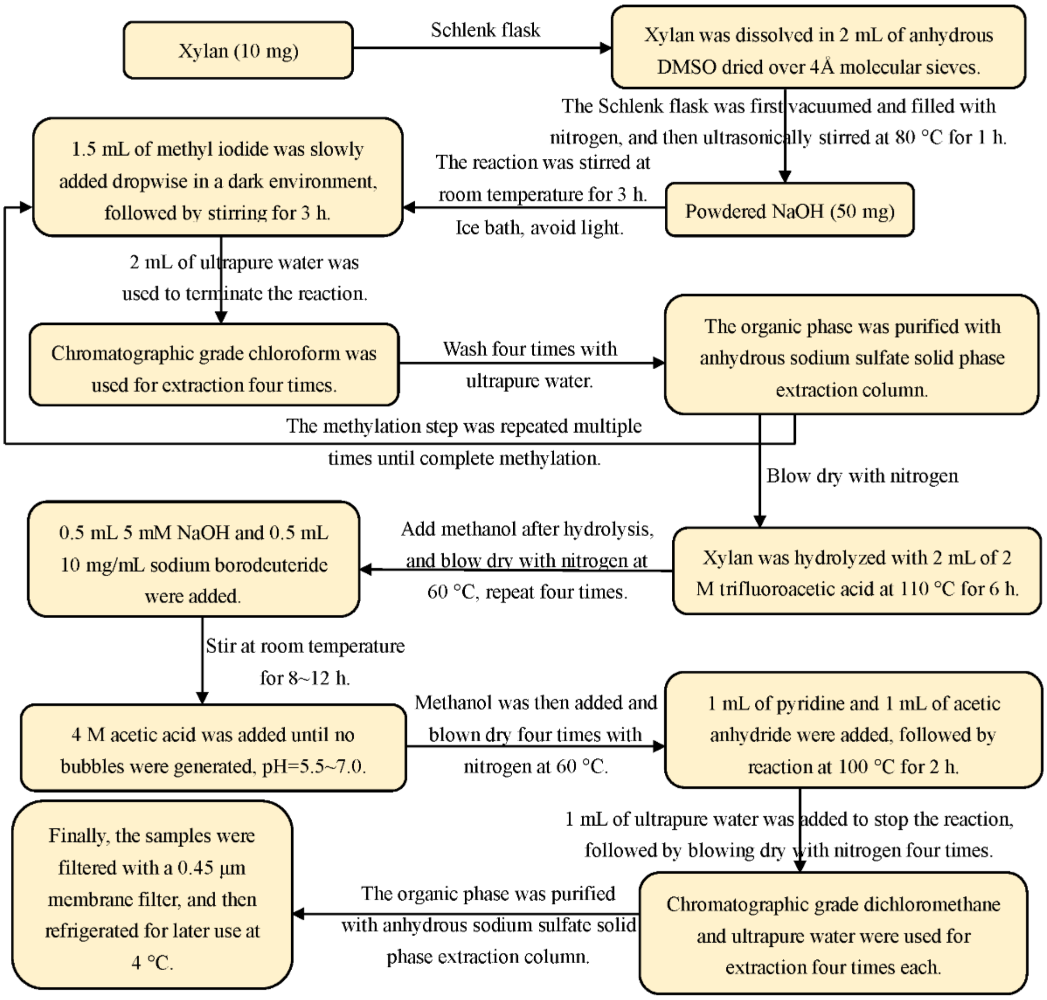

Fig.S1. The methylation treatment process of xylan

Possible DMAP-mediated sulfation of xylan with SO_3·Py

A. Donor exchange / nucleophilic activation (plausible equilibrium)

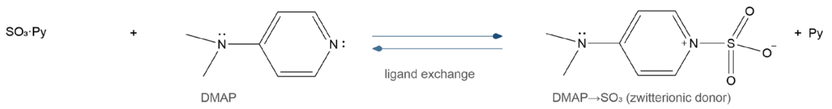

B. Sulfate transfer and catalyst regeneration

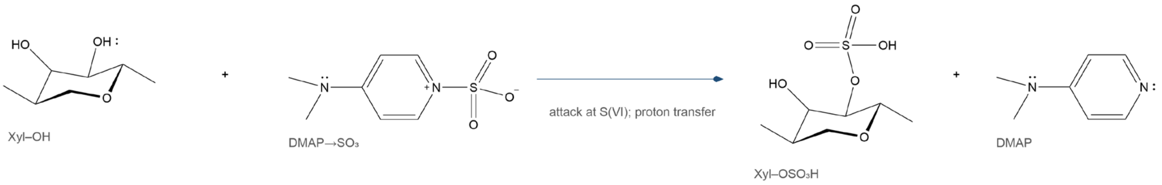

Work-up / neutralization: Xyl–OSO_3H → Xyl–OSO_3^- M^+

Interpretation: direct attack of Xyl–OH on SO_3·Py can occur in parallel. The DMAP→SO_3 species is a chemically plausible donor-exchange intermediate, not a uniquely proven resting state.

Fig. S2. Hypothetical involvement of DMAP in xylan sulfation. The proposed intermediates and regeneration steps illustrate a working hypothesis.

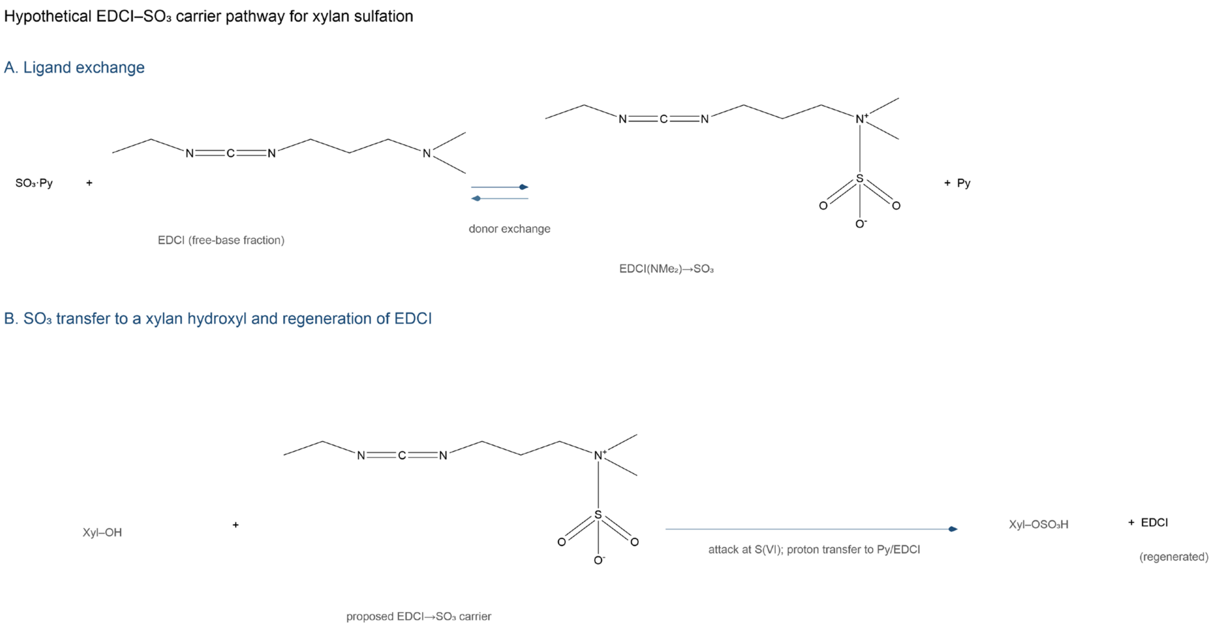

Fig. S3. Hypothetical EDCI-mediated sulfate-transfer pathway.

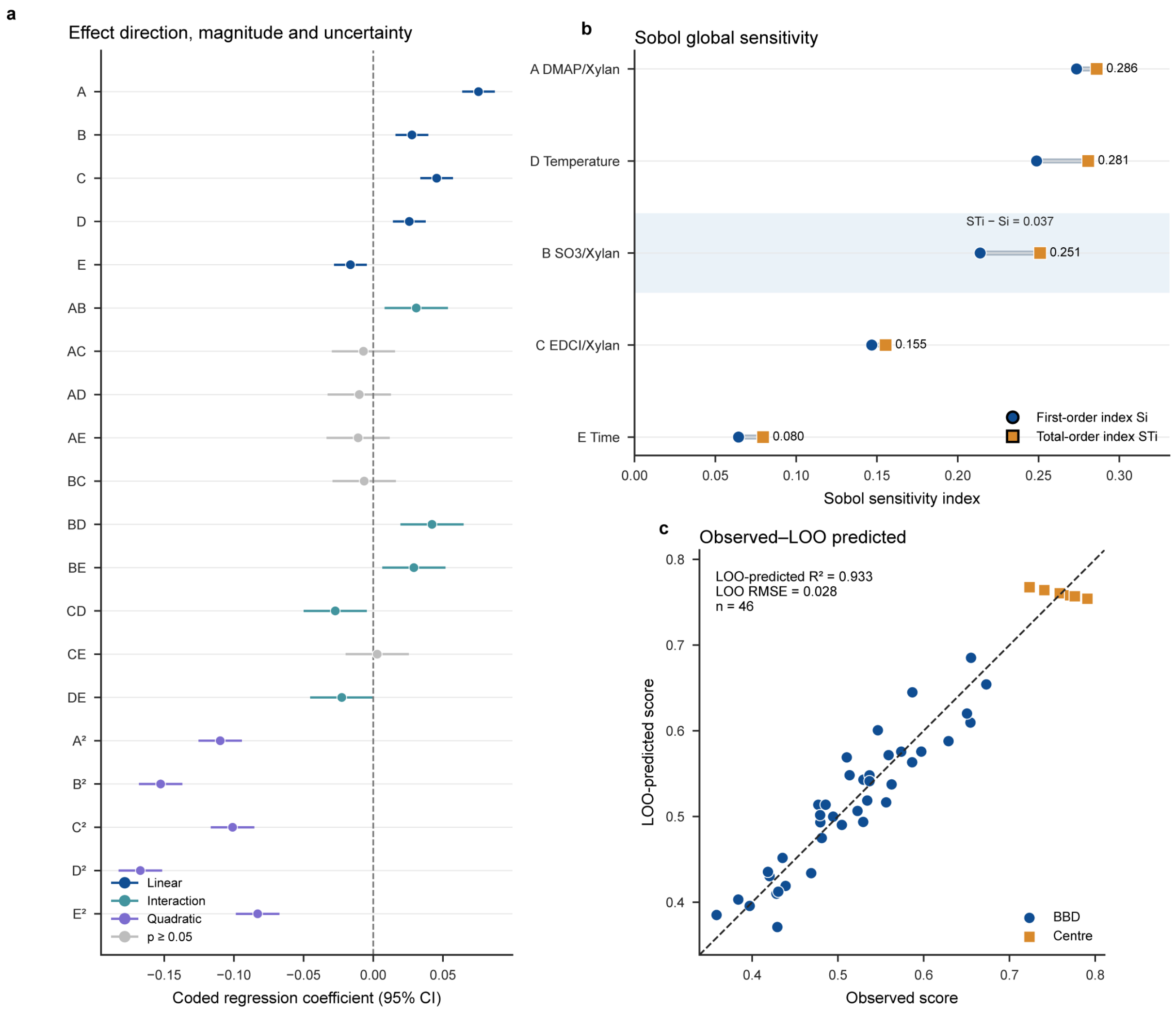

Fig. S4. Model effects, global process-factor sensitivity, and internal predictive validation of the entropy-weighted quadratic response-surface model for sugarcane-bagasse xylan sulfation. (a) Coded regression coefficients with 95% confidence intervals. Colored coefficients are significant at $p<0.05$, whereas grey coefficients are nonsignificant. (b) Exact first-order((S_i)) and total-order((S_{Ti}))$Sobo$l indices over the coded experimental domain. The difference S_{Ti}-S_i represents the contribution of interactions involving each factor. (c) Observed versus leave-one-out predicted composite sc $ores$((LOO-$predictedR^2$=0.933) , $LOORMSE$(=0.028), (n=46)). The dashed line represents identity.

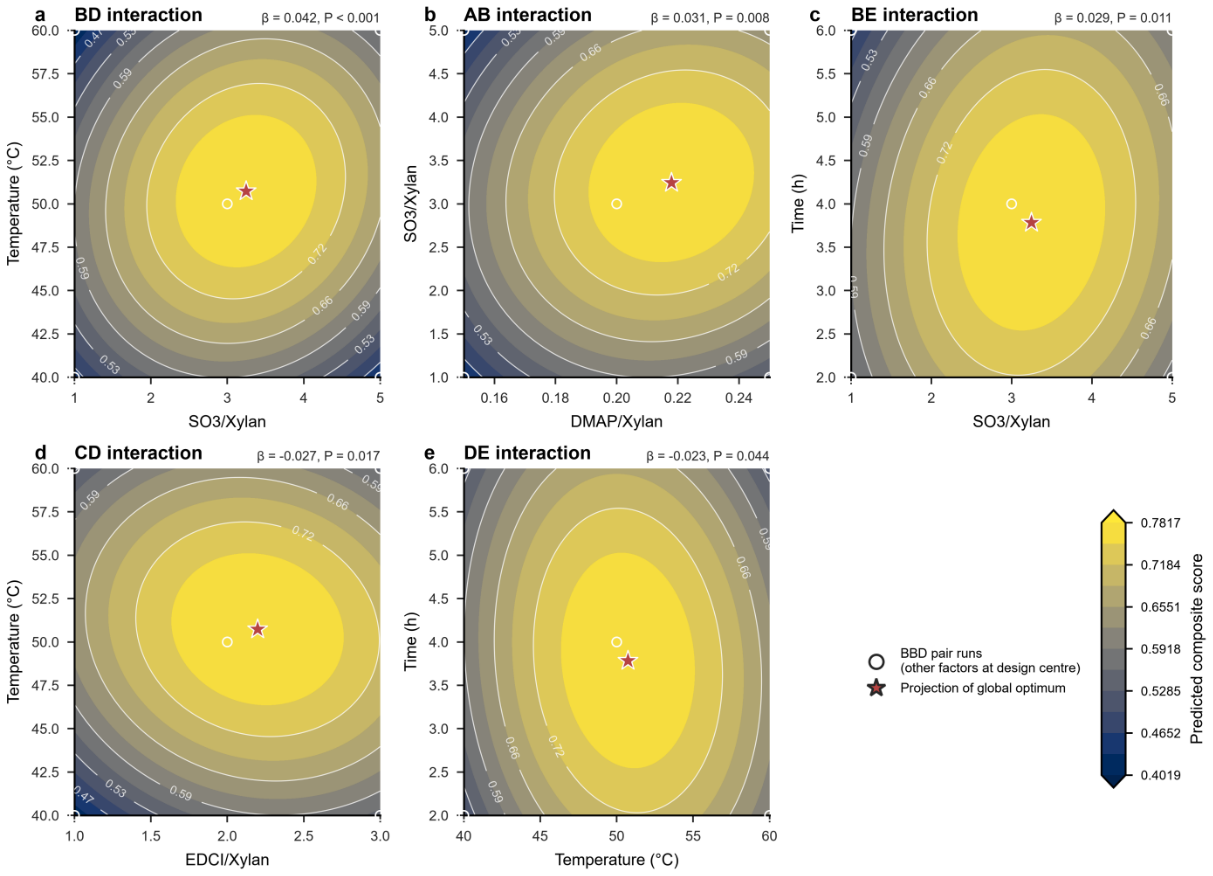

Fig. S5. Two-factor interactions governing the model-predicted composite score for the sulfation of sugarcane-bagasse xylan. Contour plots show the interactions between (a) the SO_3·Py/xylan and reaction temperature (BD), (b) the DMAP/xylan and SO_3·Py/xylan (AB), (c) the SO_3·Py/xylan and reaction time (BE), (d) the EDCI/xylan and reaction temperature (CD), and (e) reaction temperature and time (DE). The response was the entropy-weighted composite score combining normalized degree of substitution (DS; weight = 0.5696) and isolated, DS-adjusted yield (weight = 0.4304). Yellow regions indicate higher predicted scores, and white lines denote equal-score contours. The remaining three factors were fixed at the model-predicted five-dimensional global optimum: (a) A = 0.2179, C = 2.1979, and E = 3.7866 h; (b) C = 2.1979, D = 50.734 °C, and E = 3.7866 h; (c) A = 0.2179, C = 2.1979, and D = 50.734 °C; (d) A = 0.2179, B = 3.2461, and E = 3.7866 h; and (e) A = 0.2179, B = 3.2461, and C = 2.1979. The red star represents the projection of the global optimum (predicted composite score = 0.7817). Open circles indicate the corresponding Box–Behnken design runs for which the non-plotted factors were set at the design centre. β values are coded interaction coefficients from the full quadratic model, and P values test the corresponding interaction terms.

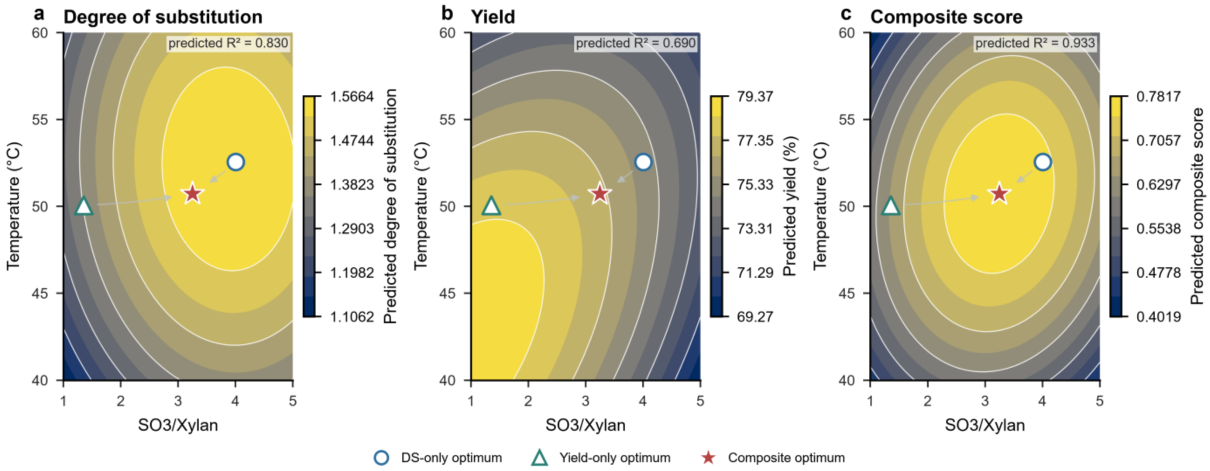

Fig. S6. Response-specific decomposition of the optimization landscape reveals an interior compromise between xylan sulfation and product recovery. Model-predicted response surfaces for (a) degree of substitution (DS), (b) DS-adjusted isolated yield, and (c) the dimensionless composite score are shown as functions of the SO_3·Py/xylan mass ratio and reaction temperature. The non-displayed factors were fixed at the composite optimum: DMAP/xylan = 0.2179, EDCI/xylan = 2.1979, and reaction time = 3.7866 h. The composite score integrates min–max-normalized DS and yield using entropy-derived weights of 0.5696 and 0.4304, respectively. The open circle, open triangle, and red star denote the B–D projections of the full five-dimensional DS-only, yield-only, and composite optima, respectively; pale arrows indicate the direction of compromise between the two response-specific objectives. Colours represent the magnitude of the fitted response, whereas white lines are iso-response contours. The reported predicted R^2 values were calculated from PRESS-based leave-one-out predictions.

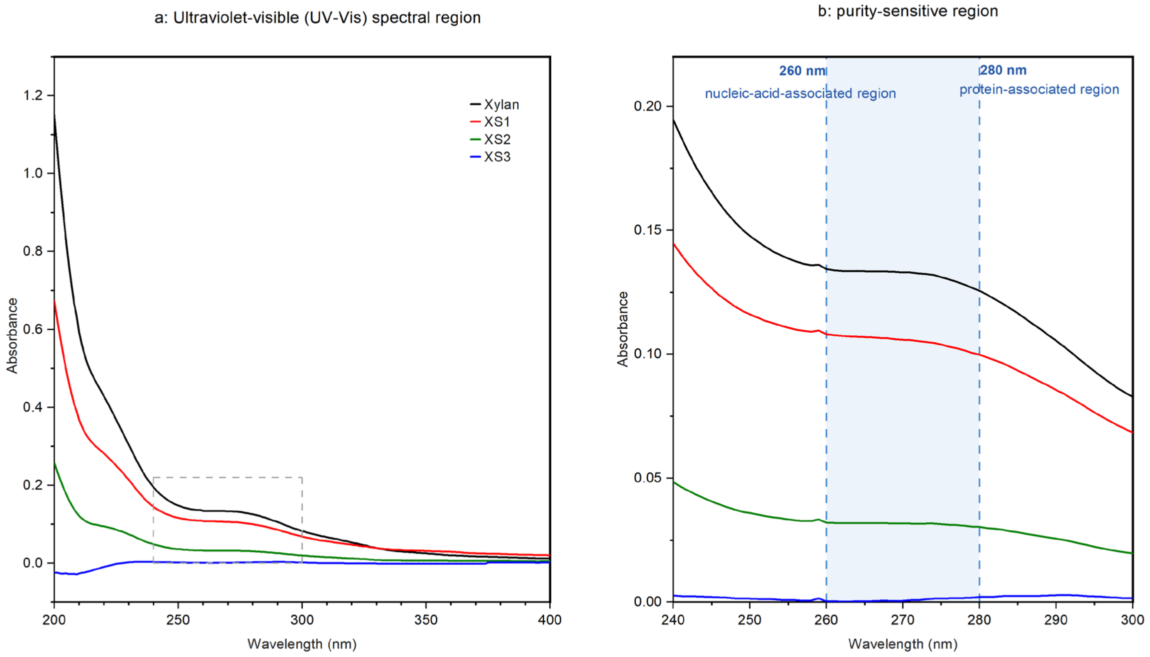

Fig.S7. UV–vis spectra of native xylan and its sulfated derivatives (XS1–XS3), measured at 2 mg/mL in ultrapure water over 200–400 nm, with ultrapure water as the reference.

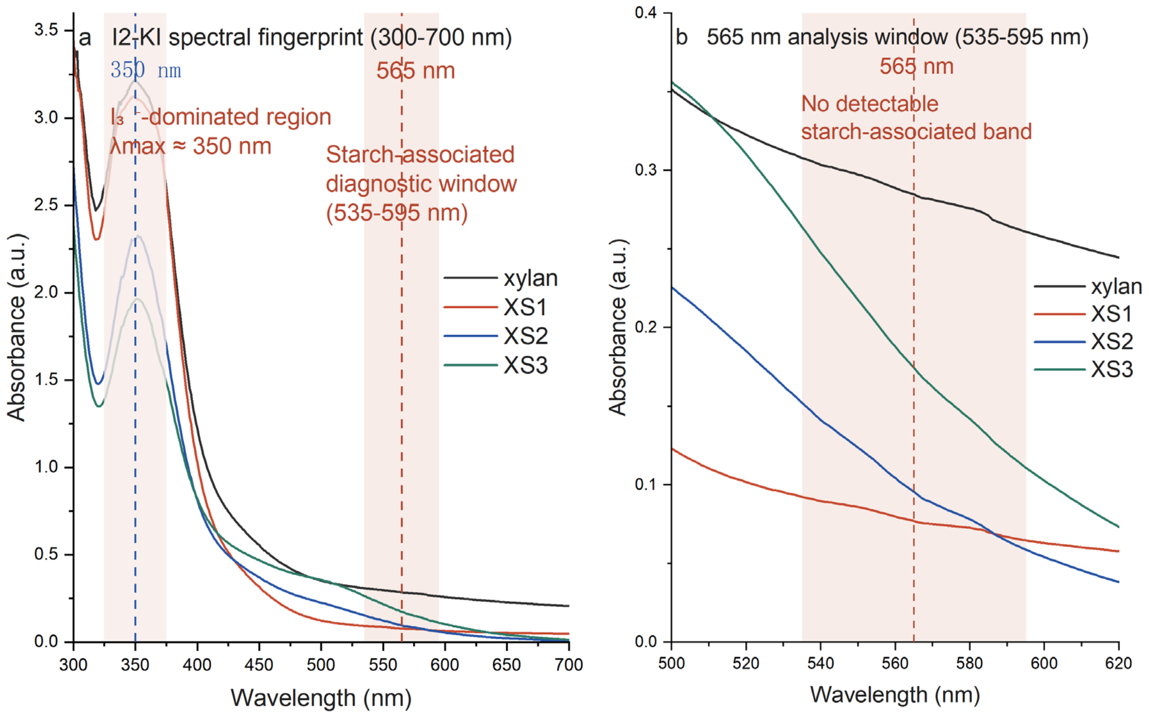

Fig.S8. UV-Vis absorption spectra of native and sulfated xylans following I_2-KI complexation.

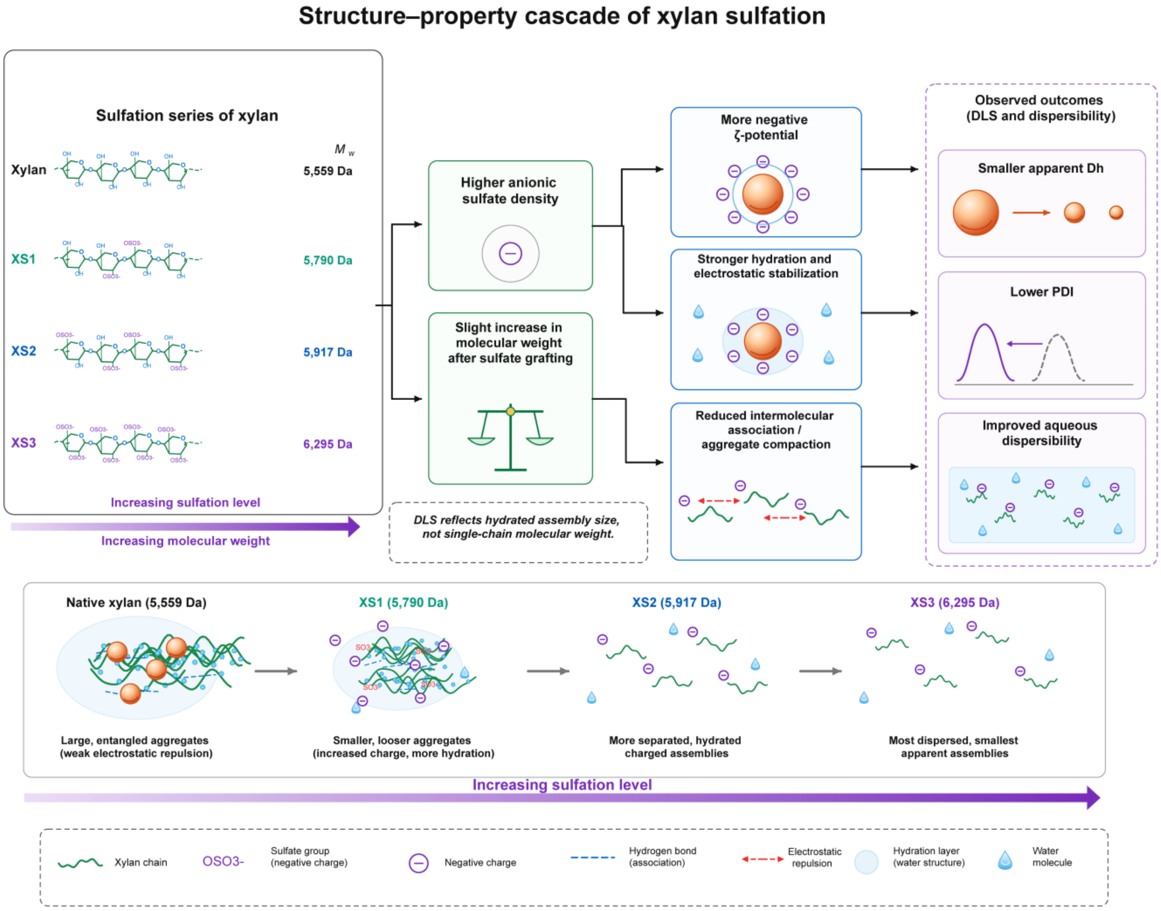

Fig.S9. Proposed structure–property cascade of xylan sulfation.
